# Mechanism of action, resistance, synergism, and clinical implications of azithromycin

**DOI:** 10.1002/jcla.24427

**Published:** 2022-04-21

**Authors:** Mohsen Heidary, Ahmad Ebrahimi Samangani, Abolfazl Kargari, Aliakbar Kiani Nejad, Ilya Yashmi, Moloudsadat Motahar, Elahe Taki, Saeed Khoshnood

**Affiliations:** ^1^ 56941 Department of Laboratory Sciences School of Paramedical Sciences Sabzevar University of Medical Sciences Sabzevar Iran; ^2^ 56941 Cellular and Molecular Research Center Sabzevar University of Medical Sciences Sabzevar Iran; ^3^ 56941 Student Research Committee Sabzevar University of Medical Sciences Sabzevar Iran; ^4^ Department of Microbiology School of Medicine Ahvaz Jundishapur University of Medical Sciences Ahvaz Iran; ^5^ 48439 Department of Microbiology School of Medicine Tehran University of Medical Sciences Tehran Iran; ^6^ 48443 Clinical Microbiology Research Center Ilam University of Medical Sciences Ilam Iran

**Keywords:** azithromycin, pharmacology, resistance, synergism, Zithromax

## Abstract

**Background:**

Azithromycin (AZM), sold under the name Zithromax, is classified as a macrolide. It has many benefits due to its immunomodulatory, anti‐inflammatory, and antibacterial effects. This review aims to study different clinical and biochemisterial aspects and properties of this drug which has a priority based on literature published worldwide.

**Methods:**

Several databases including Web of Science, Google Scholar, PubMed, and Scopus were searched to obtain the relevant studies.

**Results:**

AZM mechanism of action including the inhibition of bacterial protein synthesis, inhibition of proinflammatory cytokine production, inhibition of neutrophil infestation, and macrophage polarization alteration, gives it the ability to act against a wide range of microorganisms. Resistant organisms are spreading and being developed because of the irrational use of the drug in the case of dose and duration. AZM shows synergistic effects with other drugs against a variety of organisms. This macrolide is considered a valuable antimicrobial agent because of its use as a treatment for a vast range of diseases such as asthma, bronchiolitis, COPD, cystic fibrosis, enteric infections, STIs, and periodontal infections.

**Conclusions:**

Our study shows an increasing global prevalence of AZM resistance. Thus, synergistic combinations are recommended to treat different pathogens. Moreover, continuous monitoring of AZM resistance by registry centers and the development of more rapid diagnostic assays are urgently needed.

## Graphical abstract

Azithromycin, sold under the name Zithromax, is classified as a macrolide. It has many benefits due to its immunomodulatory, anti‐inflammatory, and antibacterial effects. Azithromycin mechanism of action including the inhibition of bacterial protein synthesis, inhibition of proinflammatory cytokine production, inhibition of neutrophil infestation, and macrophage polarization alteration, gives it the ability to act against a wide range of microorganisms.

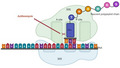

## 1 INTRODUCTION

The macrolide antibiotic AZM was developed by a group of Croatian pharmacists at PLIVA and called Sumamed, taking into account one of the great achievements in Croatia.^1^ This antibiotic was also developed under the name of Zithromax in the Pharmaceutical Chemistry Laboratories at Pfizer Central Research^2^ AZM is annually prescribed to more than 40 million patients owing to its antibacterial activity.^3^ This well‐known azalide antibiotic is structurally related to the macrolide family and can be distributed in a variety of tissues and body fluids.^4^ Due to the reversible cutting of the 50S bacterial ribosomal subunit, AZM inhibits protein synthesis and hinders the growth of bacteria.^5^, ^6^ Moreover, it can penetrate into bacterial extracellular vesicles, a kind of secretory defense system.^5^

## 2 PHARMACOLOGY

### 2.1 Pharmacodynamic of AZM

Azithromycin is classified as a macrolide antibiotic because of its unique ability.^5^ In virtue of its dual‐base structure, AZM is actively absorbed by a variety of cells, including fibroblasts and white blood cells.^7^ This antibiotic agent works in vitro against many pyogenic bacteria (e.g., *Neisseria gonorrhoeae* [*N. gonorrhoeae*] and *Moraxella catarrhalis* [*M. catarrhalis*]) and beta‐lactam‐resistant bacteria (e.g., *Legionella* and *Chlamydia* spp.).^8^ AZM has immunomodulatory, anti‐inflammatory, and antibacterial modulatory effects; thus, it is beneficial for patients with varying inflammatory diseases of the respiratory tract.^9^ AZM is also effective in patients with COVID‐19 and has been used in clinical trials for the prevention of bacterial infection in these patients. It has been reported that AZM in combination with hydroxychloroquine (HCQ) can mitigate the viral load of SARS‐CoV‐2.^10^ Moreover, AZM can modulate the features of the immune system, that is, reducing cytokine production, maintaining epithelial cell integrity, and preventing lung fibrosis.^11^ Treatment with AZM involves a short period of time. The method of its administration in adults is 1500 mg immediate‐release (IR) AZM, that is, 500 mg once daily for 3 days or 500 mg on the first day and 250 mg on Day two up to Day five.^12^ The highest oral dose approved for the treatment of gonococcal urethritis is 2.0 g of IR AZM.^12^

### 2.2 Structure of drug

**FIGURE 1:**
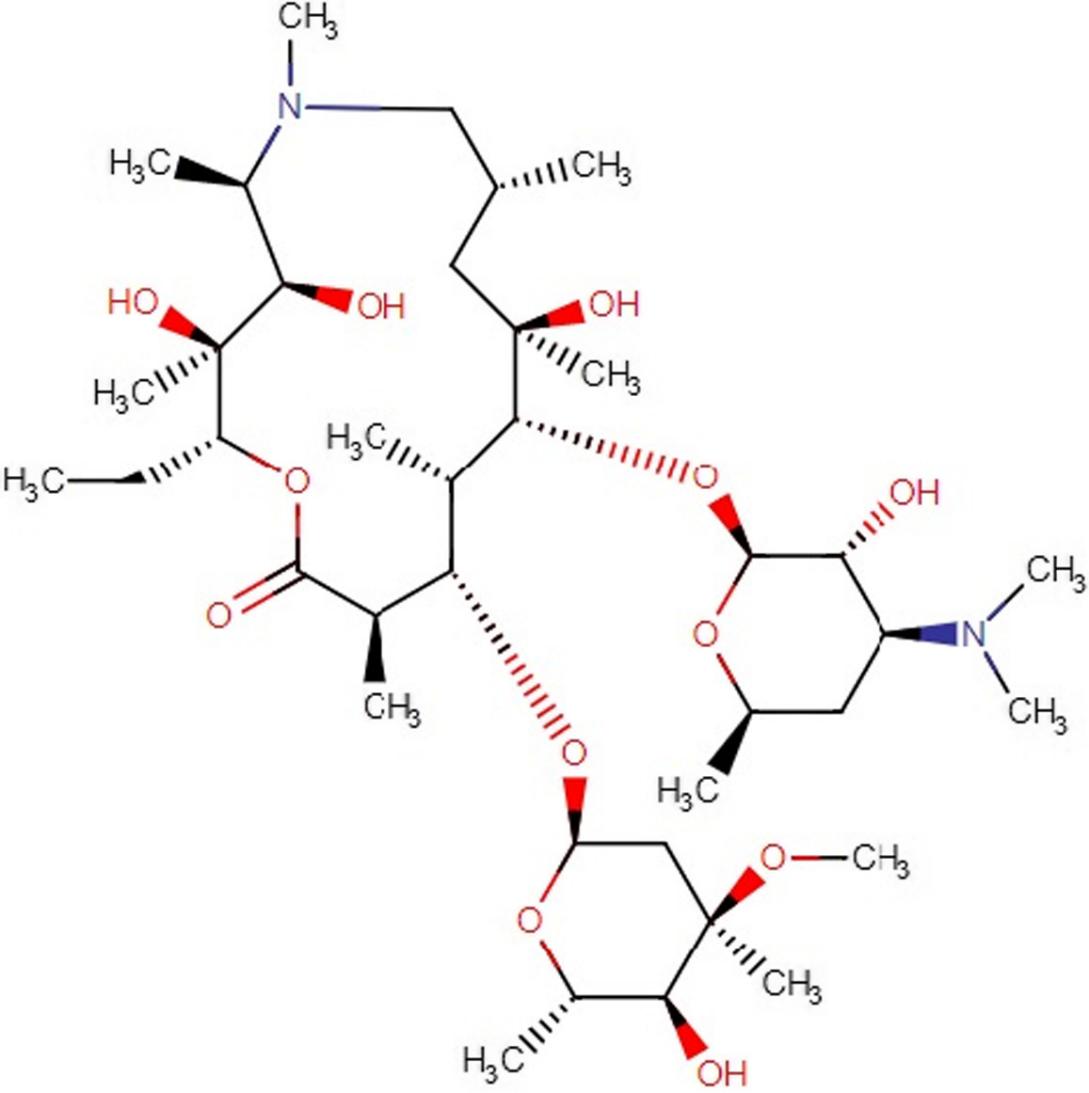
Chemical structure of azithromycin (https://go.drugbank.com/drugs/DB00207, accessed on December 18, 2021)

AZM (9‐deoxo‐9a‐methyl‐9a‐aza‐9a‐homo erythromycin A) with the chemical formula C_38_H_72_N_2_O_12_ is produced by replacing carbonyl (9a) in the aglycone ring with methyl nitrogen. Unlike erythromycin (ERY), AZM improves the durability and strength, blocks the internal reaction for hemiketal formation, and leaves the acid hydrolysis of the ether bond to the neutral sugar of L‐cladinosis, as the main decomposition pathway (Figure 1).^13^

### 2.3 Mechanism of action

**FIGURE 2:**
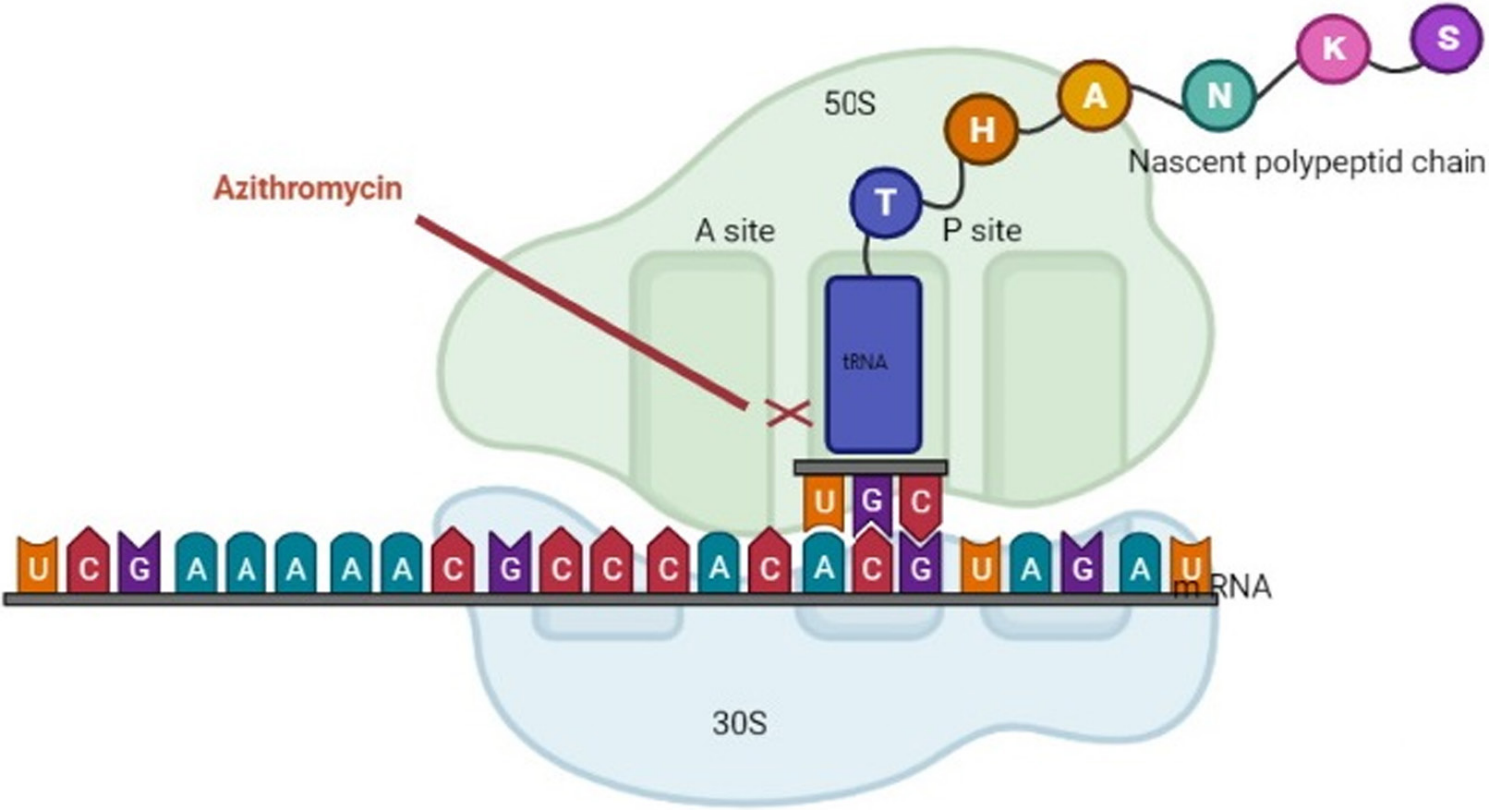
Schematic view of AZM mechanism inhibiting translation of mRNA

Similar to other macrolide antibiotics, the main objective of AZM is inhibiting bacterial protein synthesis by targeting the 50S subunit of the sensitive bacterial ribosome (Figure 2). The reduction in protein synthesis is correlated with the increase in macrolide concentration.^14^ The unionized form of AZM membrane passage rate is higher, and this could be the reason behind the increased antimicrobial activity of AZM at alkaline pH.^15^ AZM binds at a site near peptidyl transferase center on 23S rRNA called nascent peptide exit tunnel (which is approximately 100 Å long and 10–20 Å wide) and partially occludes it.^16^, ^17^ The binding process of AZM is almost similar to erythromycin. Resting of erythromycin on a surface formed by three bases (U2611, A2058, and A2059), utilizing three axial methyl groups belonging to the lactone ring of the drug is the key to this process based on research on *H. marismortui*. There is also a hydrogen bond between the 2′ OH group of the desosamine sugar of erythromycin and the N1 atom of A2058, which stabilizes erythromycin in its position. These interactions result in base movement and nascent peptide exit tunnel occlusion due to the placement of bases within van der Waals contact of the amino group of P‐site tRNA.^18^ Novel findings show that the context of the nascent peptide has an important role in changing the possibility of being allowed to pass from the peptide exit tunnel, namely AZM does not completely occlude the passage (although the nascent peptide exit tunnel has various responsibilities rather than being a normal passage to the cytoplasm such as modulating the ribosome functions in response to sequences of the novel peptide and environment).^16^ These events result in faster penetration of the outer membranes; hence, it has effects on the entrance into the bacteria and increases the activity against Gram‐negative bacteria.^17^ AZM also showed anti‐inflammatory effects on various studies; for instance, Cigana et al. demonstrated that AZM reduces TNF‐α mRNA expression, TNF‐α protein levels, and NF‐κB DNA‐binding activity in human cystic fibrosis (CF) cell lines subsequent to the confirmation of a higher rate of TNF‐α mRNA expression, TNF‐α protein levels, and NF‐κB DNA‐binding activity in CF cell lines compared with isogenic non‐CF cell lines.^19^ The reduction in NF‐κB DNA‐binding activity is associated with the inhibition of the degradation of IκBα, the protein that prohibits the translocation of NF‐κB active subunits into the nucleus.^20^ Inflammatory cell signaling is affected by AZM, and these impacts include a decrease in NF‐κB (and subsequent IL‐6 and IL‐8 production), which is mentioned above, inhibition of LPS‐induced expression of PLA2, which is involved in cytokine and chemokine production in macrophages, neutrophils, and endothelial cells and cell signaling pathways, which result in arachidonic acid and eicosanoids production, and inhibition of AP‐1 signaling in neutrophils isolated from the lungs of mice induced by LPS administration, which consequently reduce IL‐1b concentrations.^21^ AZM affects neutrophils directly and indirectly.^21^ The anti‐inflammatory properties of AZM are the reason behind the indirect effects of AZM on neutrophils. Direct effects include reduction in IL‐8 release and neutrophil airway infiltration, degranulation and degradation of extracellular myeloperoxidase, reduction in neutrophil oxidative burst,^22^, ^23^ and decrease in the production of leukotriene B4 (LTB4; a potent neutrophil chemoattractant that stimulates neutrophil IL‐8 release).^24^ AZM also helps macrophages shift from M1 type to M2 alternative‐like phenotype in vitro by inhibiting pro‐inflammatory cytokine expression (including IL‐12 and IL‐6) and shifting surface receptor expression.^25^

### 2.4 Pharmacokinetic parameters

Demethylation is the major route of metabolism, and the metabolites are not considered to have any significant antimicrobial activity.^26^ As a result of oral administration, the bioavailability of AZM reached 37%. AZM absorption may be dropped by up to 50% when administered with a large meal.^27^ AZM coadministration with aluminum‐ and magnesium‐containing antacids may reduce peak plasma concentrations by 24%, but the overall extent of absorption is not altered.^28^

The mean plasma clearance of AZM following a single 500 mg oral and intravenous dose is 630 ml/min. The primary route of AZM elimination, particularly as an unchanged drug, is through biliary excretion, and the feces are a prominent route of elimination.^26^ Moreover, over a period of 1 week, approximately 6% of the administered dose is discharged as an unchanged drug in urine; therefore, urinary excretion of AZM appears to be a minor elimination route.^26^ AZM has a half‐life of about 35–40 h in humans after a dose of 500 mg.^4^ The terminal half‐life is computed as the time required for plasma/blood concentration to decline by 50% after pseudo‐equilibrium of distribution has been accomplished. The elimination half‐life of AZM (the time for drug plasma concentration decreasing due only to drug elimination) is nearly 68 h.^29^

The long‐term studies have demonstrated AZM has no carcinogenic and mutagenic potential in standard laboratory animals and tests.^28^ The main possible adverse effects related to AZM include gastrointestinal upset, headache, dizziness, hearing loss, and cardiovascular arrhythmias. In rare cases, hepatotoxicity has been reported. In patients with a prolonged QT interval, disturbed hepatic function, and renal GFR <10 ml/min, caution should be taken when administrating AZM.^28^, ^30^

### 2.5 New formulation of AZM

A new formulation of AZM, designed as a microsphere with long‐term release (ER) to delay the release of AZM, is released slowly through bypassing the upper gastrointestinal tract after reaching its lower part. In this method, by alkalizing the formulation, elevation in the pH of the suspension minimizes the release of the drug from the microspheres in the mouth and stomach and the microsphere matrix. AZM is soluble, and this feature helps control the drug release. It spreads through the pores formed at the site of the microspheres. This ER formulation does not significantly compromise the oral bioavailability of AZM, although it bypasses a small portion of the uptake site in the upper gastrointestinal system. It achieved approximately 83% bioavailability over the IR formulation, the released microsphere formulation of AZM, allowing patients to well tolerate a full course of AZM at a dose of 2.0 g. This formulation should be taken on an empty stomach together with antacids.^31^ A new oral‐free release microsphere formulation of AZM is the first antibacterial drug approved in the USA for adult patients with mild‐to‐moderate acute bacterial sinusitis or community‐acquired pneumonia.^32^ The mentioned formulation of AZM is an oral powder that should be reconstituted with water and given in a single dose of 2.0 g. Continuous release of the drug is achieved through diffusion from the microspheres; the time to reach a peak serum concentration is 5 h. AZM is well absorbed by free release. The mean maximum serum concentration is 0.82 μg/ml, and AUC24 is about 8.62 μg/ml. Free‐release AZM should be taken on an empty stomach to ensure slower absorption. AZM is mainly excreted unchanged in feces. The final half‐life of AZM secretion is 59 h.^33^ Drug delivery to the site of infection by phagocytes and fibroblasts is characterized by tissue‐directed AZM, which provides 5‐day once‐daily diets for most infections that respond to oral therapy and 7–10 days for more serious intravenous infections. Metabolism occurs through hepatic pathways other than cytochrome P450, thus minimizing the risk of drug interactions.^8^

### 2.6 Activity in biofilms

The potential role of AZM as an antibiofilm has been studied and shown to have a planktonic state when used in aerobic conditions. It has been observed that AZM can significantly inhibit the formation and motility of biofilm in *Pseudomonas aeruginosa* (*P. aeruginosa*).^34^ Inhibition of biofilm mass in *Porphyromonas gingivalis* has also been reported among the AZM‐treated isolates.^35^ AZM in combination with Dapsone can decline the glycosaminoglycan and durability of biofilms produced by *Borrelia burgdorferi* isolates.^36^ Additionally, when combined with ciprofloxacin (CIP) or rifampin, AZM is able to completely kill the biofilm of *Bartonella henselae* within 6 days.^37^ The antibiofilm activity of the AZM pattern has also been studied among *Stenotrophomonas maltophilia* isolates and demonstrated that AZM/tigecycline combination can hamper the formation of biofilms.^38^

## 3 AZITHROMYCIN RESISTANCE

### 3.1 Mechanisms of resistance

Like other drugs, the suboptimal use of AZM has been assumed the most important cause of the development of resistant bacteria. The administration of an improper dose or duration of treatment results in the emergence and spread of resistant organisms (Table 1).^39^ Two strategies have been generally involved in gonococcal resistance against AZM: The mutations in the *mtrR* coding region resulted in overexpression of the MtrCDE efflux pump. Moreover, the affinity of *N. gonorrhoeae* to AZM decreases due to mutations in genes encoding the 23S rRNA subunit.^39^ The modification of the drug target is associated with methylation of the 23S ribosomal subunit (related to the presence of *erm* genes) or by mutations in *rrl* alleles of the 23S rRNA gene, which blocks macrolide binding to this subunit.^40^

**TABLE 1:** Mechanisms of azithromycin resistance in different bacteria

Bacteria	Mechanisms of resistance	References
*Neisseria gonorrhoeae*	(1) Over expression of an efflux pump (due to mutations at *mtrR* coding region) (2) Decreased antimicrobial affinity (due to mutations in genes encoding the 23S ribosomal subunit)	39, 40
*Pseudomonas aeruginosa*	(1) Efflux pump of *P*. *aeruginosa* confers resistance to AZM during biofilm formation (2) Mutations in the 23S rRNA gene	41, 42
Enterobacteriaceae	(1) Target mutations (23S rRNA mutations, ribosomal protein alterations) (2) Methylation (Erm‐like, Cfr‐like, RlmA‐like) (3) Decreased uptake (efflux pumps, outer membrane alterations) (4) Macrolide modification (esterases, phosphotransferases) (5) Short peptides	^43^
*Escherichia coli*	(1) Presence of chromosomal (*rplD*, *rplV*, and 23S rRNA) mutations (2) Macrolide resistance genes (MRGs) (3) Efflux pump overexpression	^44^
*Chlamydia trachomatis*	(1) Mutations of *rplD* gene, which codes for ribosomal protein L4 (2) Mutations in the peptidyl transferase region of 23S rRNA genes (3) A triple mutation in a non‐conserved region of the protein L22	^45^
*Treponema pallidum*	Mutations in 23S rRNA gene (A2058G or A2059G mutations)	^46^
*Streptococcus pneumoniae*	(1) Target modification from acquisition of the genes *erm(B)* gene by horizontal transfer (methylation by ErmB of 23S rRNA) (2) Drug efflux from the acquisition of the *mef(E)* gene by horizontal transfer (3) Chromosomal mutations in 23S rRNA genes (4) Chromosomal mutations in the genes coding for ribosomal proteins L4 or L22	^47^
*Staphylococcus aureus*	(1) Mutations in sequence of ribosomal genes *rrl* (23S rRNA) (3) Mutations in sequence of *rplV* (L22 protein)	^10^
*Salmonella*	(1) Mutations in nucleotides A2058 and A2059 of the 23S rRNA (2) Alteration of the 50S ribosomal subunit proteins L4 (*rlpD*) (3) Alteration of the 50S ribosomal subunit proteins L22 (*rlpV*)	^49^
*Haemophilus influenzae*	(1) Presence of an efflux pump homologous to the *acrAB* efflux mechanism in *E. coli* or other efflux pumps (2) L4 and L22 ribosomal protein and 23S rRNA mutations	^50^
*Legionella pneumophila*	(1) Mutations of efflux pump gene *lpeAB* (2) Mutations in genes encoding 23S rRNA or L4 and L22 ribosomal proteins	^51^
*Campylobacter*	(1) Target mutations in 23S rRNA genes (2) Target mutations in L4 and L22 ribosomal proteins (3) Ribosomal methylation encoded by *erm(B)* (4) Multidrug efflux pumps (CmeABC) (5) Decreased membrane permeability due to MOMP	52, 53

The molecular basis of the AZM resistance mechanism in *P. aeruginosa* showed that the overexpression of efflux pumps particularly mexAB‐oprM and mexCD‐oprJ^41^ and mutations in the ribosomal target of drugs in the 23S rRNA gene can cause the development of resistant strains in the biofilm community of cystic fibrosis patients.^42^ Although the better permeability and higher intracellular uptake of AZM resulted in the better activity of this antibiotic, the majority of macrolides are ineffective against Enterobacteriaceae due to intrinsic low macrolide permeability. In Enterobacteriaceae, the relevance of 23S rRNA alterations as being responsible for macrolide resistance is low since *E. coli*, *Salmonella* spp., *Shigella* spp., and *Klebsiella* spp. possess up to one or more *rrn* loci.^43^

The methylation of 23S rRNA mediated by methylases encoded in *erm* genes is the most relevant mechanism of macrolide resistance. These genes have been located in mobile elements such as plasmids carrying more than one *erm* gene.^43^, ^44^ Another type of modification related to macrolide resistance is pseudouridylation of 23S rRNA. This post‐translational modification was observed in domain V of the *E. coli* 23S rRNA. Moreover, mutations in other ribosomal proteins including L4 (encoded in the *rplD* gene) and L22 (encoded in the *rplV* gene) involved in the development of macrolide resistance in Enterobacteriaceae.^43^

It is important to note that the hydrophobic nature of macrolides has been assumed the underlying cause of intrinsic resistance to most of these antimicrobial agents. Additionally, the overexpression of chromosomal efflux pumps (AcrAB‐TolC) and outer membrane protein (OmpW) has been observed in *E. coli* AZM‐resistant mutants in vitro. In addition to mentioned mechanisms, bacterial macrolide modifications commonly lead to the decline of direct antibacterial activity.^43^ In Enterobacteriaceae, two esterases encoded by *ere* (A,B) genes and four different phosphotransferases encoded by *mph* (A, B, D, and E) hydrolyze and modify the macrolide structure.^43^, ^44^ Short peptides as 23S rRNA fragments are able to confer macrolide resistance. These resistance peptides interact with the macrolide and remove it from the ribosome and establish a new protein translation.^43^

Studies showed that mutations of the *rplD* gene contributed to less sensitive *C. trachomatis* serovar L2 isolates to AZM and ERY. It has been reported that the mutations in L4 protein conclude in the conformational modification of the 23S rRNA in domains II, III, and V resulting in disorder in the translational activity of ribosomes. Moreover, mutations in the peptidyl transferase region of 23S rRNA genes and the non‐conserved region of the protein L22 have been seen in clinical isolates resistant to *C. trachomatis*.^45^ The dramatic increase in macrolide‐resistant *Treponema pallidum (T. pallidum)* spp. *pallidum* has been reported since 2000. The emergence of macrolide resistance isolates evolves by a two‐step process including either A2058G or A2059G mutation in one copy of the 23S rRNA that subsequently results in gene conversion of both rRNA genes.^46^

The resistance mechanism of *S. pneumoniae is* associated with horizontal gene transfer of efflux pump *Mef* (E) genes. Moreover, streptococcal methylase ErmB can develop high‐level cross‐resistance to macrolides through methylation of A2058 nucleotide of 23S rRNA. Other mechanisms, including mutations in domain V of 23S rRNA and in ribosomal proteins L4 or L22, can also appear more rarely in macrolide resistance isolates of *S. pneumoniae*.^47^ The genetic mechanism of macrolide resistance of *S*. *aureus* strains isolated from cystic fibrosis patients has been well documented by mutations in genes of 23S rRNA domain II, V (*rrl*), and ribosomal protein L4 (*rplD*) and L22 (*rplV*). In addition, acquired resistance genes such as *erm* (encoding a ribosomal methylase) and *msr*(A) (encoding an efflux protein) can lead to macrolide resistance in *S. aureus* strains.^48^

Although *Salmonella* isolates have intrinsic resistance to ERY which is associated with active efflux of drugs, these strains are naturally susceptible to AZM. Resistance to macrolides is related to mutations in nucleotides A2058 and A2059 of 23S rRNA domain V. Additionally, the modification of the 50S ribosomal subunit proteins L4 and L22 may contribute to macrolide resistance.^49^ *Haemophiles influenzae* strains are intrinsically resistant to macrolide due to the presence of a homologous efflux pump to the acrAB efflux mechanism in *E. coli* or other efflux pumps. In a few strains, higher MICs related to mutations in 23S rRNA and L4 and L22 ribosomal proteins.^50^

In *Legionella pneumophila* strains, mutations in the upstream sequence of lpeAB (lpp2879–lpp2880) operon result in the overexpression of protein products. Lpp2879–Lpp2880 together with TolC forms a tripartite efflux pump of the resistance–nodulation–division (RND) family. Moreover, in AZM‐resistant isolates, mutations in 23S rRNA genes and L4/L22 ribosomal proteins have been identified.^51^ In *Campylobacter* spp., the most common mechanism for high‐level resistance to macrolides is substitutions in the domain V of the 23S rRNA gene (A2075G, A2074C/G).^52^ The substitutions and insertions in ribosomal proteins are another resistance mechanism in the absence of mutations in 23S rRNA genes. Moreover, CmeABC efflux pumps (a member of the RND transporter family) have an important role in resistance to macrolides.^52^

These three mechanisms synergistically contribute to high‐level macrolide resistance.^53^ Another mechanism of macrolide resistance in *Campylobacter* spp. is antibiotic exclusion through the major outer membrane porin (MOMP). *Campylobacter* spp. can alter membrane permeability mediated by overexpression of MOMP, chromosomally encoded by *porA*.^53^ A novel mechanism for resistance in *E. coli* isolates associated with *erm* (B) transferred by multidrug resistance (MDR) genomic islands was reported in 2014.^52^ Erm(B) methylates the 23S rRNA gene and results in decreased binding of macrolides.^52^

### 3.2 Epidemiology of resistance

#### 3.2.1 South America and Caribbean

Most published studies from America have examined the rate of AZM resistance and related mechanisms of *Shigella* spp. isolates. The resistance rate of AZM has been reported at 23.5–100% among *Shigella* spp.^54^, ^55^, ^56^ (Table 2). Although mostly *mphA* plasmid‐encoded genes were reported as determinants of reduced susceptibility to AZM in these isolates, *ermB* is identified in *Shigella* spp. isolated from men who have sex with men in Canada.^56^

**TABLE 2:** Epidemiology of azithromycin resistance

First author	Country	Enrollment time	Published time	Bacteria	No. of resistant bacteria	MIC (μg/ml)	Resistance mechanism	Resistance rate
Somani^63^	USA	1997–1998	*2000*	*Chlamydia trachomatis*	3	>4	–	–
Bhengraj^64^	India	2006–2007	*2010*	*Chlamydia trachomatis*	2	8	–	9.5%
Misyurina^65^	Russia	2000–2002	*2004*	*Chlamydia trachomatis*	4	>5.12	Mutations in a 23S rRNA and L22 Genes	66.7%
Wolter^66^	South Africa	2001–2003	*2005*	*Streptococcus pneumoniae*	2	4	Mutations in ribosomal protein L4	–
Nagai^61^	USA	1998–1999	*2000*	*Streptococcus pneumoniae*	6	16–32	*mef(E)*	50%
Gür^67^	Turkey	1996–1997	*2002*	*Streptococcus pneumoniae*	6	–	–	2.1%
Gür^67^	Turkey	1996–1997	*2002*	*Streptococcus pyogenes*	5	–	–	1.9%
Baker^68^	U.K	1995–2014	*2015*	*Shigella flexneri*	–	64–>256	Acquired antimicrobial resistance genes (pKSR100)	–
Gaudreau^54^	Canada	2012–2013	2014	*Shigella* spp.	10	≥64	*mph*(A) gene	38.5%
Sjolund Karlsson^55^	USA	2011–2012	2013	*Shigella sonnei*	4	>16	Presence of *mph*A	–
Yousfi^56^	Canada	2013–2014	2019	*Shigella* spp.	60	32–≥256	*mphA* and *ermB* genes	23.6%
Benmessaoud^69^	Morocco	2001–2012	2016	*Shigella* spp.	1	–	–	11.1%
Xiang^70^	China	2016–2018	2020	*Escherichia coli*	26	–	*mphA* gene	86.7%
Benmessaoud^69^	Morocco	2001–2012	2016	*Escherichia coli*	11	–	–	15.5%
Hoge^71^	Thailand	1995–1996	1998	*Escherichia coli*	6	>64	–	15%
Vlieghe^72^	Cambodia	2007–2010	2012	*Salmonella* spp.	20	>16	–	33.9%
Nair^73^	U.K	2012–2015	2016	*Salmonella* spp.	15	6–>16	Macrolide resistance genes (*mphA*, *mphB* or *mefB*)	2.2%
Benmessaoud^69^	Morocco	2001–2012	2016	*Salmonella* spp.	1	–	–	20%
Hoge^71^	Thailand	1995–1996	1998	*Salmonella* spp.	2	>64	–	3%
Brunner^74^	Hungary	2014–2015	2016	*Neisseria gonorrhoeae*	58	>0.5	–	30%
Kulkarni^75^	India	2013–2016	2018	*Neisseria gonorrhoeae*	6	1–8	–	5%
Cole^76^	Europe	2011–2012	2014	*Neisseria gonorrhoeae*	99	>0.5	–	5.3%
Kirkcaldy^58^	USA	2005–2013	2017	*Neisseria gonorrhoeae*	175	≥2	–	0.4%
Buder^77^	Germany	2014–2015	2018	*Neisseria gonorrhoeae*	58	≥0.5	–	10.8%
Wind^78^	Netherlands	2012–2015	2017	*Neisseria gonorrhoeae*	38	>0.5	–	1.2%
Liu^79^	Taiwan	2001–2013	2018	*Neisseria gonorrhoeae*	33	>0.5	–	14.6%
Belkacem^40^	France	2013–2014	2016	*Neisseria gonorrhoeae*	9	>0.5	Mutation in *rrl*, *mtrR*, and *rplD* genes	1%
Latif^80^	Zimbabwe	2015–2016	2018	*Neisseria gonorrhoeae*	1	4	–	10%
Dillon^57^	South America	1992–2011	2013	*Neisseria gonorrhoeae*	1114	–	–	10%
Lahra^81^	Australia	2015–2016	2016	*Neisseria gonorrhoeae*	22	–	–	1.7%
Vandepitte^82^	Uganda	2008–2009	2014	*Neisseria gonorrhoeae*	4	≥0.75	–	2.7%
Liang^83^	China	2009–2013	2016	*Neisseria gonorrhoeae*	77	≥1	Mutations in 23S rRNA, *mtrR* and *penA* genes	15.9%
Li^84^	China	2013–2015	2018	*Neisseria gonorrhoeae*	11	>1	–	3.6%
Jiang^85^	China	2014–2015	2017	*Neisseria gonorrhoeae*	36	≥1	Mutations in 23S rRNA and *mtrR* genes	28.6%
Yin^86^	China	2013–2016	2018	*Neisseria gonorrhoeae*	710	≥1	–	18.6%
Mitchell^62^	USA	2000–2004	*2006*	*Treponema pallidum*	46	–	Mutations in 23S rRNA gene	37.1%
Chen^87^	China	2008–2011	*2013*	*Treponema pallidum*	194	–	Mutations in 23S rRNA gene (A2058G mutations)	91.9%
Muldoon^88^	Ireland	2009–2010	*2012*	*Treponema pallidum*	27	–	A2058G mutations	93.1%
Vaez^89^	Iran	2018–2019	*2019*	*Haemophilus influenzae*	–	–	–	17.4%
Clark^59^	USA	2001–2002	*2002*	*Haemophilus influenzae*	10	16–>128	Mutations in 23S rRNA and ribosomal proteins L4 and L22	–
Boroumand^90^	Iran	2014–2015	*2015*	*Haemophilus influenzae*	2	–	–	10%
Peric^60^	USA	1997–2000	*2003*	*Haemophilus influenzae*	82	>4	Ribosomal mutations	1.3%
Jia^91^	China	2002–2016	*2019*	*Legionella pneumophila*	25	1.5–2	Expression of efflux pump gene *lpeAB*	16.8%
Rahimi^92^	Iran	2015–2016	*2017*	*Legionella pneumophila*	7	–	–	25.9%
Wei^93^	South Korea	2013–2016	2018	*Campylobacter* spp.	27	–	Mutation in the 23S rRNA gene	71.1%
Tang^94^	China	2019–2020	2020	*Campylobacter* spp.	62	–	Mutation in the 23S rRNA gene	66.7%
Efimochkina^95^	Russia	2019–2020	2020	*Campylobacter jejuni*	4	–	Efflux pump CmeABC genes, mutations in 23S rRNA sequence	10%
Hoge^71^	Thailand	1981–1995	1998	*Campylobacter* spp.	13	–	–	11.2%
Murphy^96^	Thailand	1994–1995	1996	*Campylobacter* spp.	9	≥8	–	31%

The gonococcal AZM susceptibility in South America and the Caribbean determined in one study examined *N*. *gonorrhoeae* isolates from 1990 to 2011. The overall prevalence of *N*. *gonorrhoeae* isolates resistant to AZM was 10.0%. Moreover, the resistance rate ranged from 25% in 2008 to 1% in 2010 in gonococcal isolates.^57^ In a Gonococcal isolate surveillance project carried out from 2005 to 2013 in the USA, the overall percentage of AZM resistance in *N*. *gonorrhoeae* isolates was 0.4% with no overall temporal trends in geometric means. These data support the continued administration of AZM in a combination therapy regimen for gonorrhea.^58^

In an in vitro evaluation of AZM resistance of 10 *Haemophilus influenzae* strains, minimum inhibitory concentrations (MICs) increased >fourfold for all strains. Mutants selected by AZM were related to alterations in 23S rRNA and ribosomal proteins L4 and L22 sequences.^59^ The macrolide susceptibility assessment of 6382 clinical *H. influenzae* isolates was studied during a 4‐year period and showed in 1.3% of the isolates, the MICs were >4 µg/ml. Among all strains that showed resistance to AZM, mutations in ribosomal proteins L4 and L22 were represented as the most common AZM resistance mechanism.^60^

A study of sequential sub‐cultures in sub‐MICs of antibiotics in 12 *S. pneumonia* strains was performed to identify resistant mutants. The overall prevalence of *S. pneumonia* isolates resistant to AZM was 50% and in all AZM‐resistant parents and derived mutants, the presence of *mefE* was reported.^61^ The molecular screening of 124 syphilis infections collected from 2000 to 2004 indicated 37.1% of *T. pallidum* isolates were resistant to AZM and associated with mutations in the 23S rRNA gene.^62^ Moreover, the first report of MDR‐resistant *Chlamydia trachomatis* (*C. trachomatis*) in the USA was reported in 2000. All three *C. trachomatis* isolates involved in this study represented a high resistance to doxycycline, AZM, and ofloxacin (OFL) (>4 µg/ml).^63^

#### 3.2.2 Asia

Several studies on the resistance of *Neisseria* isolates to AZM have been reported from East Asia. These studies examined the susceptibility of *N*. *gonorrhoeae* isolates between 2009 and 2016, and the calculated AZM resistance ranged between a high of 28.6% and 3.6% of isolates tested in China.^83^, ^84^, ^85^, ^86^ Moreover, two studies from India and Taiwan presented the overall percentage of AZM resistance in *N*. *gonorrhoeae* isolates—5% and 14.6%, respectively.^75^, ^79^ The resistance mechanism mentioned in relation to these resistant isolates was mutations in 23S rRNA, *penA*, and *mtrA* genes.^83^, ^85^ The susceptibility assessment of *Campylobacter* spp. isolated from animals and human samples carried out in South Korea, China, Russia, and Thailand.^71^, ^93^, ^94^, ^95^, ^96^

The study period ranged from 1981 to 2016 and reported that the overall percentage of AZM‐resistant *Campylobacter* spp. isolated from poultry samples differed between a high of 71.1% of isolates tested in South Korea and 10% in Russia.^93^, ^95^ Furthermore, AZM resistance among *Campylobacter* species isolated from human stool specimens was 11.2% and 31% in two studies performed in Thailand.^71^, ^96^ Frequent mutations in the 23S rRNA gene and the CmeABC efflux pump were reported as determinants of reduced susceptibility to AZM in these isolates.^93^, ^94^, ^95^ Two studies from China and Iran determined the prevalence of AZM resistance of *Legionella pneumophila* isolates 16.8% and 29.9%, respectively.^91^, ^92^ The AZM‐resistant strains were associated with overexpression levels of the efflux pump gene *lpeAB*.^91^ The AZM resistance of *Haemophilus influenzae* isolates ranged from 10% of isolates tested in 2014 increasing to 17.4% in 2018 in two studies from Iran.^89^, ^90^

A study in Russia evaluated the alteration in the 23S rRNA gene of *C*. *trachomatis* related to resistance to macrolides and reported 66.7% of *C. trachomatis* isolates were AZM‐resistant strains. The macrolide‐resistant isolates had the mutations A2058C and T2611C in the 23S rRNA gene.^65^ Moreover, in another study that examined *C*. *trachomatis* isolates obtained from recurrently infected women between 2006 and 2007, the prevalence of AZM resistance was reported at 9.5%.^64^ The antimicrobial susceptibility testing (AST) of 84 Enterotoxigenic *Escherichia coli* (ETEC) strains in China represented a high proportion of AZM resistance (86.7%) associated with a novel IncFII plasmid harboring *mphA* and *bla_TEM_*_‐1_ resistance genes.^70^ However, an overview of antibiotic susceptibility of diarrheal pathogens over a 15‐year period in Thailand demonstrated that AZM resistance was found in 15% of ETEC.^71^ Moreover, the prevalence of AZM resistance in *Salmonella* bloodstream infections in Cambodia and *T. pallidum* isolated from different areas in China reported 33.9% and 91.9, respectively.^71^, ^72^, ^87^

#### 3.2.3 Europe

Most studies reported from Europe have examined the antibiotic susceptibility of *Neisseria* isolates in different geographical areas. The results of the European gonococcal antimicrobial surveillance program published in 2011. Over a 2‐year period, 1902 *N. gonorrhoeae* isolates were collected from 21 participating countries and found that 5.3% of the examined gonococcal isolates had in vitro resistance to AZM.^76^ Between 2012 and 2015, the percentage of AZM resistance in *N. gonorrhoeae* isolated from STIs in the Netherlands reported 1.2%, and the prevalence of isolates with intermediate MICs (>0.25 and ≤0.5 mg/L) increased from 3.7% in 2012 to 8.6% in 2015.^78^

The epidemiology of AZM resistance in France during 2013–2014 indicated, among the 970 *N. gonorrhoeae* isolates, the prevalence of AZM resistance and intermediate resistance was 1% and 4.6%, respectively.^40^ The molecular analysis of isolates showed mutations in domain V of 23S rRNA, substitution and deletion in the *mtrR* promoter, and mutations in the L4 ribosomal protein associated with AZM resistance.^40^ Moreover, the antibiotic assessment of *N*. *gonorrhoeae* isolates in two separates studies accomplished during 2014–2015 reported 30% of strains collected in Hungary and 10.8% of strains collected in Germany were resistant to AZM.^74^, ^77^ A cross‐sectional study on 331 clinical isolates of *S. flexneri* serotype 3a was carried out between December 1995 and June 2014 in the UK. The strains with high‐level resistance to AZM (MIC 64 to >256 mg/L) harbored the conjugative R‐plasmid pKSR100 that carried *mphA* and *ermB*.^68^

The detection of the prevalence and mechanism of resistance to AZM of 15 isolates of non‐typhoidal *Salmonella enterica* in the UK showed 2.2% of isolates had resistance or decreased susceptibility to AZM (MIC 6 to >16 mg/L) and the presence of plasmid or chromosomally mediated genes including s *mphA*, *mphB*, and *mefB* related to AZM resistance.^73^ The molecular analysis of *T. pallidum* AZM resistance in Ireland indicated 27 out of 29 strains had the A2058G mutation and mentioned that this antibiotic should not be suggested for the treatment of syphilis in Ireland.^88^

#### 3.2.4 Africa

The assessment of antibiotic susceptibility of diarrhoeagenic bacteria collected from children in Morocco showed the prevalence of AZM resistance of *Shigella* spp., *E. coli*, and *Salmonella* spp. was 11.1%, 15.5%, and 20% respectively; however, the mechanisms involved in the antibiotic resistance of these isolates have not been identified.^69^ Moreover, the resistance rate of *N. gonorrhoeae* isolates in two studies accomplished in Uganda and Zimbabwe ranged from 2.7% in 2008–2009 to 20% in 2015–2016 respectively.^80^, ^82^ Few studies have been performed on the molecular mechanism of resistance in AZM‐resistant isolates. A study in South Africa assessed two macrolide resistance *Streptococcus pneumoniae* isolates and found the novel mechanism of resistance due to a 6 bp deletion in the gene encoding riboprotein L4.^66^

## 4 SYNERGISM

### 4.1 Synergism against *Plasmodium falciparum*

Chloroquine (CQ) is a drug that contains quinoline (a heterocyclic aromatic organic compound with the chemical formula C9H7N) and has a successful history in malaria treatment.^97^ Ohrt et al. demonstrated that CQ/AZM combination is efficacious against CQ‐resistant *Plasmodium falciparum (P. falciparum)* and AZM has an additive to synergistic activity on CQ in vitro and this therapy should be evaluated for malaria prophylaxis.^98^ In an Indian study, it was indicated that CQ/AZM combination is much more effective than AZM or CQ alone as single‐drug therapy in *P. falciparum* treatment.^99^

To discover the reason behind this synergy, Cook et al. performed a study, which revealed that synergism is not due to a systemic drug–drug interaction or the following factors: (1) the enhancement of exposure to one or both drugs because of improved bioavailability; (2) a decrease in clearance.^100^ Nakornchai et al. also support the fact that there is a range of additive to synergistic effects in CQ/AZM combination in vitro.^101^

A study was conducted by Pereira et al.^102^ which showed that CQ‐resistant isolates become more susceptible to CQ in high concentrations of AZM. They also mentioned that to achieve a maximum degree of antimalarial activity, CQ and AZM should be administered in a dose such that their potency becomes equivalent (1:1 ratio), although pediatric subjects should take a higher dose of this combination due to higher drug clearance in their body.^103^ In addition, Phiri et al. noted that CQ/AZM combination can still be a viable intermittent preventive treatment option in *P. falciparum*‐infected pregnant women in an open‐label, non‐comparative out‐patient study.^104^

A study conducted by Kshirsagar et al. in adults with acute uncomplicated *P. falciparum* malaria showed that the efficacy of CQ/AZM treatment is dependent on the dose. In other words, administration of 2 g AZM + 600 mg CQ had higher efficacy compared to 1000 or 500 mg AZM + 600 mg CQ.^105^

Quinine is also a quinoline‐containing drug that is effective against *P. falciparum*‐induced malaria.^97^ It is claimed that Quinine/AZM drug therapy is the best way to counteract MDR‐resistant *P. falciparum* in vitro.^101^ A randomized, dose‐ranging study in Thailand indicated that the combination of Quinine/AZM (quinine: 30 mg salt/kg divided three times a day and AZM: ≥1 g/day for 3 days) was effective against MDR‐resistant *P. falciparum* malaria.^106^

In a case of uncomplicated *P. falciparum* malaria, a randomized, phase 2 clinical trial was conducted in Thailand, which showed high cure rates for Quinine/AZM combination plus quinine, for a total dose of 4.5 g of AZM plus 60 mg/kg quinine or 3 days of AZM plus quinine. This study also demonstrated that AZM, which is a slow‐acting drug, should be combined with a fast‐acting drug to reach a quicker initial parasite clearance.^107^ Noedl et al. mentioned that quinine can be a promising partner for AZM. The strongest propensity toward synergy was seen in a combination ratio of 1:44.^108^

Dihydroartemisinin (DHA), an artemisinin derivative, is a drug used to treat malaria. Results showed that a 3‐day combination of dihydroartemisinin with AZM (dihydroartemisinin 80 mg or 4 tablets together with AZM 500 mg (2 capsules) for 3 days) yielded an approximately 70% cure rate, and this regimen can be a proper regimen for children and pregnant women and areas where a parasitological diagnosis is not available.^109^

### 4.2 Synergism against *Pythium insidiosum*

Pythiosis is a zoonosis disease caused by a fungus‐like pathogen, named Pythium insidiosum (*P. insidiosum*), which presents many clinical manifestations based on the type of infection.^110^ Jesus et al. demonstrated that AZM has synergistic effects with some antifungal agents such as terbinafine, amphotericin B, itraconazole, voriconazole, micafungin, caspofungin, and anidulafungin against *P. insidiosum* in vitro.^111^ Furthermore, in another study, AZM showed a synergistic effect with Carvacrol and Thymol against *P. insidiosum* in vitro.^112^

A lack of antagonism between AZM and topical drugs such as benzalkonium, cetrimide, cetylpyridinium, mupirocin, and triclosan in vitro and a lack of topical therapeutics against *P. insidiosum* suggests that these combinations may provide a potential therapy for pythiosis treatment.^113^ In vivo studies showed that AZM could be a remarkable anti‐*P. insidiosum* therapy in combination with minocycline or alone.^114^

### 4.3 Synergism against *Naegleria fowleri*

*Naegleria fowleri* is an ameba that causes a rapidly fatal infection called primary amebic meningoencephalitis (PAM) in humans. Amphotericin B, a broad‐spectrum drug, acts against most human fungal pathogens and is used to treat PAM.^115^ Soltow et al. mentioned that AZM has synergistic effects with amphotericin B against *Naegleria fowleri*; each of these drugs had less than 50% efficacy while administrated alone; however, when they were utilized with each other, the combination had 100% efficacy in vitro; therefore, it might be an acceptable regimen to treat PAM.^116^

### 4.4 Synergism against *Pseudomonas aeruginosa*

Due to increasing CIP‐resistant *P. aeruginosa* isolates, new approaches should be further investigated to treat the caused infections.^117^ Combination therapy might be the key to this subject. After the synergism of CIP and AZM was confirmed in vitro, on the peak infection day, the use of CIP/AZM combination improved clearance from the kidney and bladder and exhibited anti‐inflammatory and immunomodulatory effects in *P. aeruginosa* biofilm induced acute pyelonephritis.^118^ Saini et al. showed that this combination can also be used as a material to construct a special catheter that prevents catheter‐associated urinary tract infections in vitro.^119^

The efficacy of a novel CIP/AZM sinus stent (CASS) was evaluated in a subsequent study, and results showed that CASS delivers a sustainable amount of CIP and AZM which causes antibiofilm activity against *P. aeruginosa* in vitro.^120^ Lim et al. also demonstrated that the attainable dose of AZM released from CASS showed significant anti‐inflammatory activity by successfully reducing LPS‐stimulated IL‐8 secreted from *P. aeruginosa* in human sinonasal epithelial cells without compromising their integrity.^121^ In another study, Raouf et al. noted that CIP/AZM combination, either in free form or as nanoparticles on a chitosan nanocarrier, showed promising results including improved survival, decreased bacteriological count, and better wound healing against CIP‐resistant biofilm‐producing *P. aeruginosa* strains.^122^

### 4.5 Synergism against *Escherichia coli*

Colistin, also called polymyxin E, is a molecule that is often used as a last‐line therapy to treat MDR‐resistant Gram‐negative bacteria and can be administered either intravenously or in oral form.^123^ Li et al. demonstrated that synergistic effects were seen during a high dose of AZM administration with colistin against colistin‐resistant *Escherichia coli* isolates in vitro. Indeed, 1 or 2 mg/liter colistin + 2.5 mg/L AZM showed an eradication effect by 48 h in MZ1501R isolates in vivo.^124^

### 4.6 Synergism against Neisseria gonorrhoeae

Ceftriaxone (CRO) 250 mg intramuscular (IM) as a single dose +AZM 1 g orally as a single dose or cefixime (CFIX) 400 mg orally as a single dose + AZM 1 g orally as a single dose are suggested as dual therapies for *N. gonorrhoeae* genital or anorectal infections treatment by WHO.^125^ Furuya et al. evaluated the synergy between CFIX, cefteram (CFTM), and amoxicillin (AMX) with AZM, and the results demonstrated that CFIX/AZM combination showed a greater synergy in comparison with CFTM/AZM or amoxicillin/AZM (32% in comparison with 12% and 4%) in vitro.^126^ Onodera et al. introduced clavulanic acid/amoxicillin (CVA/AMPC) + AZM and CFTM/AZM as alternative strategies to treat CFIX‐resistant *N. gonorrhoeae*.^127^ Singh et al. noted that synergistic or additive effects were displayed in WHO‐recommended treatment without any antagonism in vitro. The absence of antagonism is the reason for the continuation of this therapy.^128^

## 5 CLINICAL TREATMENT

### 5.1 Asthma

Azithromycin accumulates in the lysosomes of phagocytic cells. In the lungs, the concentration of macrolides in neutrophils and macrophages is much higher than that measured in extracellular compartments. This information represents important cellular sites of immunomodulatory function in asthma.^129^ Hiles et al. conducted a randomized controlled trial on 420 patients to assess the effect of oral AZM in reducing the incidence of asthma. In that study, 213 patients received 500 mg of AZM and 207 received placebo three times per week. Their results showed that AZM reduced asthma exacerbations by 1.07 per patient/year, while this rate for placebo was 1.86 per patient/year. Furthermore, low‐dose AZM was an effective therapy for persistent asthma. It reduced 40% of exacerbations in severe asthma and also respiratory tract infections.^130^ In another retrospective cohort study, Douglas et al. evaluated AZM therapy in 174 children hospitalized with asthma. The overall median length of stay was 2.3 days, and 9% were readmitted for asthma within 90 days of discharge compared with 20% who had a longer length of stay after AZM treatment. Based on their results, AZM therapy was not associated with 90‐day readmission for asthma and showed no statistically significant difference in the rate of readmission in children with asthma.^131^

### 5.2 Bronchiolitis

Azithromycin is mostly used to treat lung infection and viral bronchiolitis.^132^ In a secondary analysis of a randomized double‐blinded placebo‐controlled trial, 104 infants (50 in AZM group and 54 in placebo group) were studied. That study demonstrated that 10 mg/kg of AZM can reduce the rate of recurrent wheezing, which was significantly occurred less than 6 months after discharge.^133^ By analyzing some double‐blinded placebo‐controlled studies, Che et al. evaluated the clinical efficacy of AZM adjuvant therapy in 1328 children with bronchiolitis; 667 and 661 children received AZM and placebo, respectively. The results of their study revealed that AZM could significantly diminish the time to the relief of wheezing and the detection rates of *Haemophilus influenza*, *Moraxella catarrhalis*, and *Streptococcus pneumoniae* in the nasopharyngeal region. However, no improvement was observed in the length of hospitalization and oxygen supply time.^134^

### 5.3 Chronic obstructive pulmonary disease (COPD)

Azithromycin has been shown to have the greatest effect on subjects with COPD.^135^ In a retrospective observational study, Naderi et al. randomized patients to receive AZM (250 mg, at least three times weekly for at least 6 months (*n* = 126) or neither (*n* = 69)). In AZM‐treated patients, the rate of exacerbations per patient in a year before the treatment period was 3.2, but during the following year on therapy, the rate was 2.3. In the control group, the exacerbation rates were 1.7 and 2.5 during the first and second follow‐up year, respectively. Therefore, long‐term AZM reduced the rate of exacerbation in severe COPD patients.^136^

Han et al. carried out a secondary cohort analysis study to demonstrate the effect of AZM in reducing exacerbation in COPD patients. They randomly grouped 1113 COPD patients, of which 557 and 556 subjects were received AZM and placebo, respectively. For a year, an AZM dose of 250 mg or placebo was prescribed daily. AZM was more effective than placebo in reducing COPD, although antibiotic and steroid therapy was required. The data also uncovered that AZM is more effective in older patients and patients with mild illness.^137^

In a randomized double‐blinded placebo‐controlled trial performed by Uzun et al. in the Netherlands, AZM therapy was investigated among patients with frequent exacerbations of COPD. Patients who experienced at the least three or more exacerbations in the last year received 500 mg of AZM (*n* = 47) or placebo (*n* = 45), three times a week for a year. Randomization was stratified by the use of long‐term, low‐dose prednisolone (≤10 mg daily). The number of exacerbations in the AZM group was 84 compared with placebo, which was 129. In the AZM group, the rate of exacerbations per patient per year was 1.94, while that of the placebo was 3.22. As a result, AZM can significantly reduce the exacerbation rate as compared to the placebo.^138^

### 5.4 Cystic fibrosis

Azithromycin has displayed great effects on cystic fibrosis patients. This claim was proven in a multi‐center randomized double‐blinded placebo‐controlled trial conducted by Clement et al. who assessed the long‐term effects of AZM in patients with cystic fibrosis. Their study was initiated by the randomized selection of 82 subjects who received oral AZM (*n* = 40) or placebo (*n* = 42) and ended with 35 AZM and 37 placebo cases. The patients were prescribed 250 mg or 500 mg of the mentioned agents depending on their body weight, three times a week for 12 months. The results showed that the rate of pulmonary exacerbations, the time elapsed before the first pulmonary exacerbation, and the number of additional courses of oral antibiotics declined in the AZM group, but not in the placebo group.^139^

### 5.5 Enteric infections

Due to increasing drug‐resistant Enterobacteriaceae, AZM is needed to treat enteric infections. An open‐labeled, non‐comparative study was carried out by Aggarwal et al. to uncover the effect of AZM on the treatment of uncomplicated typhoid fever. In their investigation, 109 children received daily 20 mg of AZM per kg for 6 days; 102 patients were cured completely.^140^ In another study, patients with uncomplicated typhoid fever received intravenous CRO (75 mg/day, *n* = 36) or AZM (20 mg/kg/day, *n* = 32) for 5 days. Although the result did not show a significant difference between AZM and CRO groups, treatment with AZM did not have any relapse, and they had a longer time to clearance of bacteremia. Thus, AZM can be an appropriate treatment for children with typhoid fever.^141^

Similarly, in a randomized controlled study, Parry et al. compared AZM with OFL for treating 187 patients with MDR typhoid fever. Patients were randomly categorized into three groups to receive AZM (10 mg/day/kg, *n* = 62), OFL (20 mg/day/kg, *n* = 63), or their combination (10 mg/day/kg AZM in days one to three and 15 mg/day/kg OFL for 7 days, *n* = 62). The results showed that AZM alone may be a better choice to cure uncomplicated typhoid fever, and it has a shorter treatment duration.^142^

In another prospective randomized trial, Vukelic et al. compared AZM with ERY in the treatment of children infected with *Campylobacter concisus*. They randomized 120 patients into four groups, including ERY (50 mg/day/kg for 5 days), AZM (a single dose of 20 mg/kg or 30 mg/kg), and control who received no treatment. Results indicated that 30 mg/kg AZM was more efficient in curing *Campylobacter enterocolitis* in children. Moreover, they proved that this effect was dose dependent.^143^

### 5.6 Sexually transmitted infections

Azithromycin has been shown to be highly efficient in bacterial STIs caused by *C. trachomatis*, *N. gonorrhoeae*, and *T. pallidum*.^144^ Recently, Macaux et al. performed a retrospective cohort study on the effect of AZM on the treatment of *C. trachomatis*‐infected patients. They evaluated the efficiency of a single dose of AZM oral administration in 50 patients with asymptomatic rectal infection. The overall results demonstrated that treatment with AZM can be effective against asymptomatic rectal *C. trachomatis* infection.^145^

### 5.7 Periodontal infections

Due to the penetration of periodontal infections in deep tissue or inaccessible areas, viz., the tooth furcation or gingival tissues, the function of AZM in treating these infections becomes more important. AZM concentrates on neutrophils, macrophages, and fibroblasts and plays a key role in fighting periodontal disease.^146^

In a randomized trial, Mascarenhas et al. selected 31 patients who smoked more than a pack daily and investigated the effect of AZM in combination with scaling and root planning (SRP) for treating severe chronic periodontitis.

Patients were randomly given SRP alone or SRP + AZM. The results indicated a clinical improvement in both groups within 6 months of treatment; however, the combination group indicated more reduction rate in probing depths and clinical attachment loss and deep sites.^147^

## 6 CONCLUSIONS

Azithromycin is a semisynthetic macrolide that has a significant effect on a wide range of Gram‐positive and Gram‐negative bacteria. Both alone and/or in synergy with other antibiotics, AZM has been used successfully for the treatment of respiratory diseases (such as asthma, bronchiolitis, COPD, and cystic fibrosis), enteric infections, periodontal infections, and STDs. However, pharmacokinetics/pharmacodynamics studies have demonstrated an incomplete absorption of this antibiotic with low oral bioavailability. Various bacteria in different countries have shown different levels of antibiotic resistance to AZM. Thus, in this study, we reviewed the mechanisms and epidemiology of AZM resistance worldwide. Overall, the data show that the global prevalence of AZM resistance is increasing among bacteria. Resistance to AZM is developing similar to many other drugs; therefore, synergistic combinations are prescribed and being studied to confront different pathogens. Therefore, it is necessary to discover the AZM mechanism of action and the underlying mechanism behind the synergism with different drugs that effectively act against different organisms. A great variety of combinations could be studied in various outlooks including synergism and effects on the human body and different AZM combinations are no exception. Therefore, continuous monitoring of AZM resistance by AST methods, the establishment of an antibiotic resistance registry center, using electronically reporting systems, and the development of more rapid diagnostic assays are recommended.

## CONFLICT OF INTEREST

The authors report no conflicts of interest in this work.

## AUTHOR CONTRIBUTIONS

S.K., M.H., and E.T. involved in study concept and design. M.H., A.E.S., A.K., A.k.N., I.Y., M.M., E.T., and S.K. drafted the article. S.K., M.H., and E.T. involved in critical revision of the article for important intellectual content. S.K. and E.T involved in study supervision. All the authors have read and agreed to the published version of the article.

## References

[1] Tomišić Z . The story of azithromycin. Kem Ind J Chem Chem Eng. 2011;60:603‐617.

[2] Retsema J , Girard A , Schelkly W , et al. Spectrum and mode of action of azithromycin (CP‐62,993), a new 15‐membered‐ring macrolide with improved potency against gram‐negative organisms. Antimicrob Agents Chemother. 1987;31:1939‐1947. doi:10.1128/aac.31.12.1939 2449865 PMC175832

[3] Mosholder AD , Mathew J , Alexander JJ , Smith H , Nambiar S . Cardiovascular risks with azithromycin and other antibacterial drugs. N Engl J Med. 2013;368:1665‐1668. doi:10.1056/NEJMp1302726 23635046

[4] Amsden GW . Erythromycin, clarithromycin, and azithromycin: are the differences real? Clin Ther. 1996;18:56‐72. 8851453 10.1016/s0149-2918(96)80179-2

[5] Imamura Y , Higashiyama Y , Tomono K , et al. Azithromycin exhibits bactericidal effects on Pseudomonas aeruginosa through interaction with the outer membrane. Antimicrob Agents Chemother. 2005;49:1377‐1380. doi:10.1128/aac.49.4.1377-1380.2005 15793115 PMC1068619

[6] Jelić D , Antolović R . From erythromycin to azithromycin and new potential ribosome‐binding antimicrobials. Antibiotics. 2016;5:29. doi:10.3390/antibiotics5030029 PMC5039525 27598215

[7] Gladue RP , Snider ME . Intracellular accumulation of azithromycin by cultured human fibroblasts. Antimicrob Agents Chemother. 1990;34:1056. doi:10.1128/AAC.34.6.1056 2168141 PMC171758

[8] Rapp RP . Pharmacokinetics and pharmacodynamics of intravenous and oral azithromycin: enhanced tissue activity and minimal drug interactions. Ann Pharmacother. 1998;32:785‐793. doi:10.1345/aph.17299 9681095

[9] Albert RK , Connett J , Bailey WC , et al. Azithromycin for prevention of exacerbations of COPD. N Engl J Med. 2011;365:689‐698. doi:10.1056/NEJMoa1104623 21864166 PMC3220999

[10] Gautret P , Lagier JC , Parola P , et al. Hydroxychloroquine and azithromycin as a treatment of COVID‐19: results of an open‐label non‐randomized clinical trial. Int J Antimicrob Agents. 2020;56:105949. doi:10.1016/j.ijantimicag.2020.105949 32205204 PMC7102549

[11] Horowitz RI , Freeman PR . Precision medicine: retrospective chart review and data analysis of 200 patients on dapsone combination therapy for chronic Lyme disease/post‐treatment Lyme disease syndrome: part 1. Int J Gen Med. 2019;12:101‐119. doi:10.2147/ijgm.S193608 30863136 PMC6388746

[12] Liu P , Allaudeen H , Chandra R , et al. Comparative pharmacokinetics of azithromycin in serum and white blood cells of healthy subjects receiving a single‐dose extended‐release regimen versus a 3‐day immediate‐release regimen. Antimicrob Agents Chemother. 2007;51:103. doi:10.1128/AAC.00852-06 17060516 PMC1797671

[13] Padayachee N , Schellack N . Focus on azithromycin. S Afr Gen Pract. 2021;2:6‐8.

[14] Champney WS , Burdine R . Azithromycin and clarithromycin inhibition of 50S ribosomal subunit formation in *Staphylococcus aureus* cells. Curr Microbiol. 1998;36:119‐123. 9425251 10.1007/s002849900290

[15] Bakheit A , Al‐Hadiya B , Abd‐Elgalil A . Azithromycin. Profiles Drug Subst Excip Relat Methodol. 2014;39:1‐40. 24794904 10.1016/B978-0-12-800173-8.00001-5

[16] Vázquez‐Laslop N , Mankin AS . How macrolide antibiotics work. Trends Biochem Sci. 2018;43:668‐684. 30054232 10.1016/j.tibs.2018.06.011 PMC6108949

[17] Parnham MJ , Haber VE , Giamarellos‐Bourboulis EJ , Perletti G , Verleden GM , Vos R . Azithromycin: mechanisms of action and their relevance for clinical applications. Pharmacol Ther. 2014;143:225‐245. 24631273 10.1016/j.pharmthera.2014.03.003

[18] Bulkley D , Innis CA , Blaha G , Steitz TA . Revisiting the structures of several antibiotics bound to the bacterial ribosome. Proc Natl Acad Sci USA. 2010;107:17158‐17163. 20876130 10.1073/pnas.1008685107 PMC2951403

[19] Cigana C , Assael BM , Melotti P . Azithromycin selectively reduces tumor necrosis factor alpha levels in cystic fibrosis airway epithelial cells. Antimicrob Agents Chemother. 2007;51:975‐981. 17210769 10.1128/AAC.01142-06 PMC1803122

[20] Aghai ZH , Kode A , Saslow JG , et al. Azithromycin suppresses activation of nuclear factor‐kappa B and synthesis of pro‐inflammatory cytokines in tracheal aspirate cells from premature infants. Pediatric Res. 2007;62:483‐488. 10.1203/PDR.0b013e318142582d 17667842

[21] Venditto VJ , Haydar D , Abdel‐Latif A , et al. Immunomodulatory effects of azithromycin revisited: potential applications to COVID‐19. Front Immunol. 2021;12:285. 10.3389/fimmu.2021.574425 PMC7906979 33643308

[22] Verleden GM , Vanaudenaerde BM , Dupont LJ , Van Raemdonck DE . Azithromycin reduces airway neutrophilia and interleukin‐8 in patients with bronchiolitis obliterans syndrome. Am J Respir Crit Care Med. 2006;174:566‐570. 16741151 10.1164/rccm.200601-071OC

[23] Čulić O , Eraković V , Čepelak I , et al. Azithromycin modulates neutrophil function and circulating inflammatory mediators in healthy human subjects. Eur J Pharmacol. 2002;450:277‐289. 12208321 10.1016/s0014-2999(02)02042-3

[24] Wales D , Woodhead M . The anti‐inflammatory effects of macrolides. Thorax. 1999;54:S58. 10451695 10.1136/thx.54.2008.s58 PMC1765933

[25] Kannan K , Mankin AS . Macrolide antibiotics in the ribosome exit tunnel: species‐specific binding and action. Ann N Y Acad Sci. 2011;1241:33‐47. 22191525 10.1111/j.1749-6632.2011.06315.x

[26] Lode H . The pharmacokinetics of azithromycin and their clinical significance. Eur J Clin Microbiol Infect Dis. 1991;10:807‐812. 1662623 10.1007/BF01975832

[27] Fohner AE , Sparreboom A , Altman RB , Klein TE . PharmGKB summary: macrolide antibiotic pathway, pharmacokinetics/pharmacodynamics. Pharm Genom. 2017;27:164. 10.1097/FPC.0000000000000270 PMC5346035 28146011

[28] Drew RH , Gallis HA . Azithromycin—spectrum of activity, pharmacokinetics, and clinical applications. Pharmacother J Hum Pharmacol Drug Ther. 1992;12:161‐173. 1319048

[29] Nahata MC , Koranyi K , Gadgil S , Hilligoss D , Fouda H , Gardner M . Pharmacokinetics of azithromycin in pediatric patients after oral administration of multiple doses of suspension. Antimicrob Agents Chemother. 1993;37:314‐316. 8383944 10.1128/aac.37.2.314 PMC187659

[30] McMullan BJ , Mostaghim M . Prescribing azithromycin. Aust Prescr. 2015;38:87. 26648627 10.18773/austprescr.2015.030 PMC4653965

[31] Chandra R , Liu P , Breen JD , et al. Clinical pharmacokinetics and gastrointestinal tolerability of a novel extended‐release microsphere formulation of azithromycin. Clin Pharmacokinet. 2007;46:247‐259. doi:10.2165/00003088-200746030-00005 17328583

[32] Swainston Harrison T , Keam SJ . Azithromycin extended release: a review of its use in the treatment of acute bacterial sinusitis and community‐acquired pneumonia in the US. Drugs. 2007;67:773‐792. doi:10.2165/00003495-200767050-00010 17385947

[33] Harrison TS , Keam SJ . Azithromycin extended release. Drugs. 2007;67:773‐792. doi:10.2165/00003495-200767050-00010 17385947

[34] Lozano C , López M , Rojo‐Bezares B , Sáenz Y . Antimicrobial susceptibility testing in *Pseudomonas aeruginosa* biofilms: one step closer to a standardized method. Antibiotics. 2020;9:880. doi:10.3390/antibiotics9120880 PMC7763878 33316877

[35] Anggani HS , Perdana RG , Siregar E , Bachtiar EW . The effect of coating chitosan on *Porphyromonas gingivalis* biofilm formation in the surface of orthodontic mini‐implant. J Adv Pharm Technol Res. 2021;12:84‐88. doi:10.4103/japtr.JAPTR_95_20 33532361 PMC7832189

[36] Horowitz RI , Murali K , Gaur G , Freeman PR , Sapi E . Effect of dapsone alone and in combination with intracellular antibiotics against the biofilm form of *B. burgdorferi* . BMC Res Notes. 2020;13:455. doi:10.1186/s13104-020-05298-6 32993780 PMC7523330

[37] Zheng X , Ma X , Li T , Shi W , Zhang Y . Effect of different drugs and drug combinations on killing stationary phase and biofilms recovered cells of Bartonella henselae in vitro. BMC Microbiol. 2020;20:87. doi:10.1186/s12866-020-01777-9 32276590 PMC7149919

[38] Yue C , Shen W , Hu L , et al. Effects of tigecycline combined with azithromycin against biofilms of multidrug‐resistant Stenotrophomonas maltophilia isolates from a patient in China. Infect Drug Resist. 2021;14:775‐786. doi:10.2147/idr.S298274 33679134 PMC7924117

[39] Derbie A , Mekonnen D , Woldeamanuel Y , Abebe T . Azithromycin resistant gonococci: a literature review. Antimicrob Resist Infect Control. 2020;9:1‐7. 32811545 10.1186/s13756-020-00805-7 PMC7436955

[40] Belkacem A , Jacquier H , Goubard A , et al. Molecular epidemiology and mechanisms of resistance of azithromycin‐resistant Neisseria gonorrhoeae isolated in France during 2013–14. J Antimicrob Chemother. 2016;71:2471‐2478. 27301565 10.1093/jac/dkw182

[41] Gillis RJ , White KG , Choi KH , Wagner VE , Schweizer HP , Iglewski BH . Molecular basis of azithromycin‐resistant *Pseudomonas aeruginosa* biofilms. Antimicrob Agents Chemother. 2005;49:3858‐3867. doi:10.1128/aac.49.9.3858-3867.2005 16127063 PMC1195439

[42] Chalmers JD . Macrolide resistance in *Pseudomonas aeruginosa*: implications for practice. Eur Respir Soc. 2017;49:1700689. 10.1183/13993003.00689-2017 28526802

[43] Gomes C , Martínez‐Puchol S , Palma N , et al. Macrolide resistance mechanisms in Enterobacteriaceae: focus on azithromycin. Crit Rev Microbiol. 2017;43:1‐30. 27786586 10.3109/1040841X.2015.1136261

[44] Gomes C , Ruiz‐Roldán L , Mateu J , Ochoa TJ , Ruiz J . Azithromycin resistance levels and mechanisms in *Escherichia coli* . Sci Rep. 2019;9:1‐10. 30988366 10.1038/s41598-019-42423-3 PMC6465286

[45] Mestrovic T , Ljubin‐Sternak S . Molecular mechanisms of Chlamydia trachomatis resistance to antimicrobial drugs. Front Biosci. 2018;23:656‐670. 10.2741/4611 28930567

[46] Šmajs D , Paštěková L , Grillová L . Macrolide resistance in the syphilis spirochete, Treponema pallidum ssp. pallidum: can we also expect macrolide‐resistant yaws strains? Am J Trop Med Hyg. 2015;93:678‐683. 26217043 10.4269/ajtmh.15-0316 PMC4596581

[47] Gingras H , Patron K , Leprohon P , Ouellette M . Azithromycin resistance mutations in Streptococcus pneumoniae as revealed by a chemogenomic screen. Microb Genom. 2020;6:454. 10.1099/mgen.0.000454 PMC7725334 33074087

[48] Prunier A‐L , Malbruny B , Laurans M , Brouard J , Duhamel J‐F , Leclercq R . High rate of macrolide resistance in *Staphylococcus aureus* strains from patients with cystic fibrosis reveals high proportions of hypermutable strains. J Infect Dis. 2003;187:1709‐1716. 12751028 10.1086/374937

[49] Gunell M , Kotilainen P , Jalava J , Huovinen P , Siitonen A , Hakanen AJ . In vitro activity of azithromycin against nontyphoidal Salmonella enterica. Antimicrob Agents Chemother. 2010;54:3498‐3501. 20498312 10.1128/AAC.01678-09 PMC2916314

[50] Tristram S , Jacobs MR , Appelbaum PC . Antimicrobial resistance in Haemophilus influenzae. Clin Microbiol Rev. 2007;20:368‐389. 17428889 10.1128/CMR.00040-06 PMC1865592

[51] Massip C , Descours G , Ginevra C , Doublet P , Jarraud S , Gilbert C . Macrolide resistance in Legionella pneumophila: the role of LpeAB efflux pump. J Antimicrob Chemother. 2017;72:1327‐1333. 28137939 10.1093/jac/dkw594

[52] Bolinger H , Kathariou S . The current state of macrolide resistance in Campylobacter spp.: trends and impacts of resistance mechanisms. Appl Environ Microbiol. 2017;83:e00416‐e00417. 28411226 10.1128/AEM.00416-17 PMC5452823

[53] Iovine NM . Resistance mechanisms in *Campylobacter jejuni* . Virulence. 2013;4:230‐240. 23406779 10.4161/viru.23753 PMC3711981

[54] Gaudreau C , Barkati S , Leduc J‐M , Pilon PA , Favreau J , Bekal S . Shigella spp. with reduced azithromycin susceptibility, Quebec, Canada, 2012–2013. Emerg Infect Dis. 2014;20:854. 24750584 10.3201/eid2005.130966 PMC4012797

[55] Sjölund Karlsson M , Bowen A , Reporter R , et al. Outbreak of infections caused by *Shigella sonnei* with reduced susceptibility to azithromycin in the United States. Antimicrob Agents Chemother. 2013;57:1559‐1560. doi:10.1128/AAC.02360-12 23274665 PMC3591876

[56] Yousfi K , Gaudreau C , Pilon PA , et al. Genetic mechanisms behind the spread of reduced susceptibility to azithromycin in Shigella strains isolated from men who have sex with men in Québec, Canada. Antimicrob Agents Chemother. 2019;63:e01679‐18. doi:10.1128/aac.01679-18 30455248 PMC6355565

[57] Dillon J‐AR , Trecker MA , Thakur SD . Two decades of the gonococcal antimicrobial surveillance program in South America and the Caribbean: challenges and opportunities. Sex Transm Infect. 2013;89:iv36‐iv41. 24243878 10.1136/sextrans-2012-050905

[58] Kirkcaldy RD , Bartoces MG , Soge OO , et al. Antimicrobial drug prescription and Neisseria gonorrhoeae susceptibility, United States, 2005‐2013. Emerg Infect Dis. 2017;23:1657‐1663. doi:10.3201/eid2310.170488 28930001 PMC5621530

[59] Clark C , Bozdogan BI , Peric M , Dewasse B , Jacobs MR , Appelbaum PC . In vitro selection of resistance in Haemophilus influenzae by amoxicillin‐clavulanate, cefpodoxime, cefprozil, azithromycin, and clarithromycin. Antimicrob Agents Chemother. 2002;46:2956‐2962. 12183253 10.1128/AAC.46.9.2956-2962.2002 PMC127454

[60] Peric M , Bozdogan BI , Jacobs MR , Appelbaum PC . Effects of an efflux mechanism and ribosomal mutations on macrolide susceptibility of Haemophilus influenzae clinical isolates. Antimicrob Agents Chemother. 2003;47:1017‐1022. 12604536 10.1128/AAC.47.3.1017-1022.2003 PMC149331

[61] Nagai K , Davies TA , Dewasse BE , Pankuch GA , Jacobs MR , Appelbaum PC . In vitro development of resistance to ceftriaxone, cefprozil and azithromycin in Streptococcus pneumoniae. J Antimicrob Chemother. 2000;46:909‐915. 11102409 10.1093/jac/46.6.909

[62] Mitchell SJ , Engelman J , Kent CK , Lukehart SA , Godornes C , Klausner JD . Azithromycin‐resistant syphilis infection: San Francisco, California, 2000–2004. Clin Infect Dis off Publ Infect Dis Soc Am. 2006;42:337‐345. doi:10.1086/498899 16392078

[63] Somani J , Bhullar VB , Workowski KA , Farshy CE , Black CM . Multiple drug‐resistant Chlamydia trachomatis associated with clinical treatment failure. J Infect Dis. 2000;181:1421‐1427. 10762573 10.1086/315372

[64] Bhengraj AR , Vardhan H , Srivastava P , Salhan S , Mittal A . Decreased susceptibility to azithromycin and doxycycline in clinical isolates of Chlamydia trachomatis obtained from recurrently infected female patients in India. Chemotherapy. 2010;56:371‐377. 20938174 10.1159/000314998

[65] Misyurina O , Chipitsyna E , Finashutina Y , et al. Mutations in a 23S rRNA gene of Chlamydia trachomatis associated with resistance to macrolides. Antimicrob Agents Chemother. 2004;48:1347‐1349. 15047540 10.1128/AAC.48.4.1347-1349.2004 PMC375314

[66] Wolter N , Smith AM , Farrell DJ , et al. Novel mechanism of resistance to oxazolidinones, macrolides, and chloramphenicol in ribosomal protein L4 of the pneumococcus. Antimicrob Agents Chemother. 2005;49:3554‐3557. 16048983 10.1128/AAC.49.8.3554-3557.2005 PMC1196237

[67] Gür D , Ozalp M , Sümerkan B , et al. Prevalence of antimicrobial resistance in Haemophilus influenzae, Streptococcus pneumoniae, Moraxella catarrhalis and Streptococcus pyogenes: results of a multicentre study in Turkey. Int J Antimicrob Agents. 2002;19:207‐211. doi:10.1016/s0924-8579(02)00003-1 11932143

[68] Baker KS , Dallman TJ , Ashton PM , et al. Intercontinental dissemination of azithromycin‐resistant shigellosis through sexual transmission: a cross‐sectional study. Lancet Infect Dis. 2015;15:913‐921. 25936611 10.1016/S1473-3099(15)00002-X

[69] Benmessaoud R , Nezha M , Moraleda C , et al. Antimicrobial resistance levels among diarrhoeagenic micro‐organisms recovered from children under‐5 with acute moderate‐to‐severe diarrhoea in Rabat, Morocco. J Glob Antimicrob Resist. 2016;7:34‐36. 27568103 10.1016/j.jgar.2016.07.005

[70] Xiang Y , Wu F , Chai Y , et al. A new plasmid carrying mphA causes prevalence of azithromycin resistance in enterotoxigenic *Escherichia coli* serogroup O6. BMC Microbiol. 2020;20:1‐9. 32782021 10.1186/s12866-020-01927-z PMC7418381

[71] Hoge CW , Gambel JM , Srijan A , Pitarangsi C , Echeverria P . Trends in antibiotic resistance among diarrheal pathogens isolated in Thailand over 15 years. Clin Infect Dis. 1998;26:341‐345. 9502453 10.1086/516303

[72] Vlieghe ER , Phe T , De Smet B , et al. Azithromycin and ciprofloxacin resistance in Salmonella bloodstream infections in Cambodian adults. PLoS Negl Trop Dis. 2012;6:e1933. 23272255 10.1371/journal.pntd.0001933 PMC3521708

[73] Nair S , Ashton P , Doumith M , et al. WGS for surveillance of antimicrobial resistance: a pilot study to detect the prevalence and mechanism of resistance to azithromycin in a UK population of non‐typhoidal Salmonella. J Antimicrob Chemother. 2016;71:3400‐3408. 27585964 10.1093/jac/dkw318

[74] Brunner A , Nemes‐Nikodem E , Jeney C , et al. Emerging azithromycin‐resistance among the Neisseria gonorrhoeae strains isolated in Hungary. Ann Clin Microbiol Antimicrob. 2016;15:1‐6. 27646968 10.1186/s12941-016-0166-9 PMC5029006

[75] Kulkarni SV , Bala M , Muqeeth SA , et al. Antibiotic susceptibility pattern of Neisseria gonorrhoeae strains isolated from five cities in India during 2013–2016. J Med Microbiol. 2018;67:22‐28. 29231153 10.1099/jmm.0.000662

[76] Cole M , Spiteri G , Chisholm S , et al. Emerging cephalosporin and multidrug‐resistant gonorrhoea in Europe. Eurosurveillance. 2014;19:20955. 25411689 10.2807/1560-7917.es2014.19.45.20955

[77] Buder S , Dudareva S , Jansen K , et al. Antimicrobial resistance of Neisseria gonorrhoeae in Germany: low levels of cephalosporin resistance, but high azithromycin resistance. BMC Infect Dis. 2018;18:1‐11. 29343220 10.1186/s12879-018-2944-9 PMC5772720

[78] Wind CM , van der Loeff MFS , van Dam AP , de Vries HJ , van der Helm JJ . Trends in antimicrobial susceptibility for azithromycin and ceftriaxone in Neisseria gonorrhoeae isolates in Amsterdam, the Netherlands, between 2012 and 2015. Eurosurveillance. 2017;22:30431. 28079519 10.2807/1560-7917.ES.2017.22.1.30431 PMC5388096

[79] Liu Y‐H , Huang Y‐T , Liao C‐H , Hsueh P‐R . Antimicrobial susceptibilities and molecular typing of Neisseria gonorrhoeae isolates at a medical centre in Taiwan, 2001–2013 with an emphasis on high rate of azithromycin resistance among the isolates. Int J Antimicrob Agents. 2018;51:768‐774. 29477549 10.1016/j.ijantimicag.2018.01.024

[80] Latif AS , Gwanzura L , Machiha A , et al. Antimicrobial susceptibility in Neisseria gonorrhoeae isolates from five sentinel surveillance sites in Zimbabwe, 2015–2016. Sex Transm Infect. 2018;94:62‐66. doi:10.1136/sextrans-2016-053090 28476914

[81] Lahra MM , Enriquez RP . Australian gonococcal surveillance programme, 1 July to 30 September 2015. Commun Dis Intell Q Rep. 2016;40:E179‐E181. 27080026 10.33321/cdi.2016.40.8

[82] Vandepitte J , Hughes P , Matovu G , Bukenya J , Grosskurth H , Lewis DA . High prevalence of ciprofloxacin‐resistant gonorrhea among female sex workers in Kampala, Uganda (2008–2009). Sex Transm Dis. 2014;41:233‐237. 24622633 10.1097/OLQ.0000000000000099

[83] Liang J‐Y , Cao W‐L , Li X‐D , et al. Azithromycin‐resistant Neisseria gonorrhoeae isolates in Guangzhou, China (2009–2013): coevolution with decreased susceptibilities to ceftriaxone and genetic characteristics. BMC Infect Dis. 2016;16:1‐8. 27080231 10.1186/s12879-016-1469-3 PMC4832481

[84] Li W , Zhu BY , Qin SQ , et al. Surveillance of antibiotic susceptibility patterns of Neisseria gonorrhoeae from 2013 to 2015 in Guangxi Province, China. Jpn J Infect Dis. 2018;71:148‐151. doi:10.7883/yoken.JJID.2017.169 29279442

[85] Jiang F‐X , Lan Q , Le W‐J , Su X‐H . Antimicrobial susceptibility of Neisseria gonorrhoeae isolates from Hefei (2014‐2015): genetic characteristics of antimicrobial resistance. BMC Infect Dis. 2017;17:1‐6. 28545411 10.1186/s12879-017-2472-z PMC5445337

[86] Yin Y‐P , Han Y , Dai X‐Q , et al. Susceptibility of Neisseria gonorrhoeae to azithromycin and ceftriaxone in China: a retrospective study of national surveillance data from 2013 to 2016. PLoS Med. 2018;15:e1002499. 29408881 10.1371/journal.pmed.1002499 PMC5800545

[87] Chen X‐S , Yin Y‐P , Wei W‐H , et al. High prevalence of azithromycin resistance to Treponema pallidum in geographically different areas in China. Clin Microbiol Infect. 2013;19:975‐979. 23231450 10.1111/1469-0691.12098

[88] Muldoon EG , Walsh A , Crowley B , Mulcahy F . Treponema pallidum azithromycin resistance in Dublin, Ireland. Sex Transm Dis. 2012;39:784‐786. 23001265 10.1097/OLQ.0b013e318269995f

[89] Vaez H , Sahebkar A , Pourfarzi F , Yousefi‐Avarvand A , Khademi F . Prevalence of antibiotic resistance of Haemophilus Influenzae in Iran‐a meta‐analysis. Iran J Otorhinolaryngol. 2019;31:349. 31857979 10.22038/ijorl.2019.34363.2137 PMC6914328

[90] Boroumand M , Irani S , Siadat SD , Bouzari S . Molecular detection of genomic islands associated with class 1 and 2 integron in Haemophilus influenzae isolated in Iran. Jundishapur J Microbiol. 2015;8:e17249. 26034545 10.5812/jjm.8(4)2015.17249 PMC4449856

[91] Jia X , Ren H , Nie X , Li Y , Li J , Qin T . Antibiotic resistance and azithromycin resistance mechanism of Legionella pneumophila serogroup 1 in China. Antimicrob Agents Chemother. 2019;63:e00768‐19. 31405864 10.1128/AAC.00768-19 PMC6761501

[92] Rahimi B , Vesal A . Antimicrobial resistance properties of legionella pneumophila isolated from the cases of lower respiratory tract infections. Biomed Pharmacol J. 2017;10:59‐65.

[93] Wei B , Kang M . Molecular basis of macrolide resistance in Campylobacter strains isolated from poultry in South Korea. BioMed Res Int. 2018;2018:4526576. 30069469 10.1155/2018/4526576 PMC6057423

[94] Tang M , Zhou Q , Zhang X , et al. Antibiotic resistance profiles and molecular mechanisms of Campylobacter from chicken and pig in China. Front Microbiol. 2020;11:2722. 10.3389/fmicb.2020.592496 PMC7652819 33193261

[95] Efimochkina N , Stetsenko V , Sheveleva S . Formation of the resistance of *Campylobacter jejuni* to macrolide antibiotics. Bull Exp Biol Med. 2020;169:351‐356. 32748135 10.1007/s10517-020-04885-8

[96] Murphy GS Jr , Echeverria P , Jackson LR , Arness MK , LeBron C , Pitarangsi C . Ciprofloxacin‐and azithromycin‐resistant Campylobacter causing traveler's diarrhea in US troops deployed to Thailand in 1994. Clin Infect Dis. 1996;22:868‐869. 8722958 10.1093/clinids/22.5.868

[97] Slater AF . Chloroquine: mechanism of drug action and resistance in Plasmodium falciparum. Pharmacol Ther. 1993;57:203‐235. doi:10.1016/0163-7258(93)90056-j 8361993

[98] Ohrt C , Willingmyre GD , Lee P , Knirsch C , Milhous W . Assessment of azithromycin in combination with other antimalarial drugs against Plasmodium falciparum in vitro. Antimicrob Agents Chemother. 2002;46:2518‐2524. doi:10.1128/aac.46.8.2518-2524.2002 12121927 PMC127390

[99] Dunne MW , Singh N , Shukla M , et al. A multicenter study of azithromycin, alone and in combination with chloroquine, for the treatment of acute uncomplicated Plasmodium falciparum malaria in India. J Infect Dis. 2005;191:1582‐1588. doi:10.1086/429343 15838784

[100] Cook JA , Randinitis EJ , Bramson CR , Wesche DL . Lack of a pharmacokinetic interaction between azithromycin and chloroquine. Am J Trop Med Hyg. 2006;74:407‐412. 16525098

[101] Nakornchai S , Konthiang P . Activity of azithromycin or erythromycin in combination with antimalarial drugs against multidrug‐resistant Plasmodium falciparum in vitro. Acta Trop. 2006;100:185‐191. doi:10.1016/j.actatropica.2006.10.008 17126280

[102] Pereira MR , Henrich PP , Sidhu AB , et al. In vivo and in vitro antimalarial properties of azithromycin‐chloroquine combinations that include the resistance reversal agent amlodipine. Antimicrob Agents Chemother. 2011;55:3115‐3124. doi:10.1128/aac.01566-10 21464242 PMC3122405

[103] Zhao Q , Tensfeldt TG , Chandra R , Mould DR . Population pharmacokinetics of azithromycin and chloroquine in healthy adults and paediatric malaria subjects following oral administration of fixed‐dose azithromycin and chloroquine combination tablets. Malar J. 2014;13:36. doi:10.1186/1475-2875-13-36 24472224 PMC3909452

[104] Phiri K , Kimani J , Mtove GA , et al. Parasitological clearance rates and drug concentrations of a fixed dose combination of azithromycin‐chloroquine in asymptomatic pregnant women with Plasmodium falciparum parasitemia: an open‐label, non‐comparative study in Sub‐Saharan Africa. PLoS One. 2016;11:e0165692. doi:10.1371/journal.pone.0165692 27861509 PMC5115659

[105] Kshirsagar NA , Gogtay NJ , Moran D , et al. Treatment of adults with acute uncomplicated malaria with azithromycin and chloroquine in India, Colombia, and Suriname. Res Rep Trop Med. 2017;8:85‐104. doi:10.2147/rrtm.s129741 30050349 PMC6038897

[106] Miller RS , Wongsrichanalai C , Buathong N , et al. Effective treatment of uncomplicated Plasmodium falciparum malaria with azithromycin‐quinine combinations: a randomized, dose‐ranging study. Am J Trop Med Hyg. 2006;74:401‐406. 16525097

[107] Noedl H , Krudsood S , Chalermratana K , et al. Azithromycin combination therapy with artesunate or quinine for the treatment of uncomplicated Plasmodium falciparum malaria in adults: a randomized, phase 2 clinical trial in Thailand. Clin Infect Dis off Publ Infect Dis Soc Am. 2006;43:1264‐1271. doi:10.1086/508175 17051490

[108] Noedl H , Krudsood S , Leowattana W , et al. In vitro antimalarial activity of azithromycin, artesunate, and quinine in combination and correlation with clinical outcome. Antimicrob Agents Chemother. 2007;51:651‐656. doi:10.1128/aac.01023-06 17116669 PMC1797729

[109] Krudsood S , Buchachart K , Chalermrut K , et al. A comparative clinical trial of combinations of dihydroartemisinin plus azithromycin and dihydroartemisinin plus mefloquine for treatment of multidrug resistant falciparum malaria. Southeast Asian J Trop Med Public Health. 2002;33:525‐531. 12693587

[110] Chitasombat MN , Jongkhajornpong P , Lekhanont K , Krajaejun T . Recent update in diagnosis and treatment of human pythiosis. PeerJ. 2020;8:e8555. doi:10.7717/peerj.8555 32117626 PMC7036273

[111] Jesus FP , Ferreiro L , Loreto ÉS , et al. In vitro synergism observed with azithromycin, clarithromycin, minocycline, or tigecycline in association with antifungal agents against Pythium insidiosum. Antimicrob Agents Chemother. 2014;58:5621‐5625. doi:10.1128/aac.02349-14 25001300 PMC4135807

[112] Jesus FP , Ferreiro L , Bizzi KS , et al. In vitro activity of carvacrol and thymol combined with antifungals or antibacterials against Pythium insidiosum. J Mycol Med. 2015;25:e89‐e93. doi:10.1016/j.mycmed.2014.10.023 25639921

[113] Itaqui SR , Verdi CM , Tondolo JS , et al. In vitro synergism between azithromycin or terbinafine and topical antimicrobial agents against Pythium insidiosum. Antimicrob Agents Chemother. 2016;60:5023‐5025. doi:10.1128/aac.00154-16 27216049 PMC4958184

[114] Jesus FP , Loreto ÉS , Ferreiro L , et al. In vitro and in vivo antimicrobial activities of minocycline in combination with azithromycin, clarithromycin, or tigecycline against Pythium insidiosum. Antimicrob Agents Chemother. 2016;60:87‐91. doi:10.1128/aac.01480-15 26459895 PMC4704155

[115] Warnock DW . Amphotericin B: an introduction. J Antimicrob Chemother. 1991;28:27‐38. doi:10.1093/jac/28.suppl_b.27 1778890

[116] Soltow SM , Brenner GM . Synergistic activities of azithromycin and amphotericin B against Naegleria fowleri in vitro and in a mouse model of primary amebic meningoencephalitis. Antimicrob Agents Chemother. 2007;51:23‐27. doi:10.1128/aac.00788-06 17060522 PMC1797677

[117] Rehman A , Patrick WM , Lamont IL . Mechanisms of ciprofloxacin resistance in *Pseudomonas aeruginosa*: new approaches to an old problem. J Med Microbiol. 2019;68:1‐10. doi:10.1099/jmm.0.000873 30605076

[118] Saini H , Chhibber S , Harjai K . Azithromycin and ciprofloxacin: a possible synergistic combination against Pseudomonas aeruginosa biofilm‐associated urinary tract infections. Int J Antimicrob Agents. 2015;45:359‐367. doi:10.1016/j.ijantimicag.2014.11.008 25604277

[119] Saini H , Chhibber S , Harjai K . Antimicrobial and antifouling efficacy of urinary catheters impregnated with a combination of macrolide and fluoroquinolone antibiotics against *Pseudomonas aeruginosa* . Biofouling. 2016;32:511‐522. doi:10.1080/08927014.2016.1155564 26982572

[120] Lim DJ , Skinner D , McLemore J , et al. In‐vitro evaluation of a ciprofloxacin and azithromycin sinus stent for *Pseudomonas aeruginosa* biofilms. Int Forum Allergy Rhinol. 2020;10:121‐127. doi:10.1002/alr.22475 31692289 PMC6942221

[121] Lim DJ , Thompson HM , Walz CR , et al. Azithromycin and ciprofloxacin inhibit interleukin‐8 secretion without disrupting human sinonasal epithelial integrity in vitro. Int Forum Allergy Rhinol. 2021;11:136‐143. doi:10.1002/alr.22656 32725797 PMC7854841

[122] Raouf M , Essa S , El Achy S , Essawy M , Rafik S , Baddour M . Evaluation of combined Ciprofloxacin and azithromycin free and nano formulations to control biofilm producing Pseudomonas aeruginosa isolated from burn wounds. Indian J Med Microbiol. 2021;39:81‐87. doi:10.1016/j.ijmmb.2021.01.004 33460732

[123] Grégoire N , Aranzana‐Climent V , Magréault S , Marchand S , Couet W . Clinical pharmacokinetics and pharmacodynamics of Colistin. Clin Pharmacokinet. 2017;56:1441‐1460. doi:10.1007/s40262-017-0561-1 28550595

[124] Li Y , Lin X , Yao X , et al. Synergistic antimicrobial activity of Colistin in combination with rifampin and azithromycin against *Escherichia coli* producing MCR‐1. Antimicrob Agents Chemother. 2018;62:e01631‐18. doi:10.1128/aac.01631-18 PMC6256761 30224527

[125] WHO Guidelines Approved by the Guidelines Review Committee . WHO Guidelines for the Treatment of Neisseria Gonorrhoeae. World Health Organization©. 2016. 27512795

[126] Furuya R , Nakayama H , Kanayama A , et al. In vitro synergistic effects of double combinations of beta‐lactams and azithromycin against clinical isolates of Neisseria gonorrhoeae. J Infect Chemother off J Jpn Soc Chemother. 2006;12:172‐176. doi:10.1007/s10156-006-0445-z 16944253

[127] Onodera S , Kiyota H , Endo K , et al. Enhancement of antimicrobial activities of cefteram or clavulanic acid/amoxicillin against cefixime‐resistant Neisseria gonorrhoeae in the presence of clarithromycin or azithromycin. J Infect Chemother off J Jpn Soc Chemother. 2006;12:207‐209. doi:10.1007/s10156-006-0447-x 16944260

[128] Singh V , Bala M , Bhargava A , Kakran M , Bhatnagar R . In vitro efficacy of 21 dual antimicrobial combinations comprising novel and currently recommended combinations for treatment of drug resistant gonorrhoea in future era. PLoS One. 2018;13:e0193678. doi:10.1371/journal.pone.0193678 29509792 PMC5839552

[129] Fricker M , Gibson PG . Macrophage dysfunction in the pathogenesis and treatment of asthma. Eur Respir J. 2017;50:1700196. 28899935 10.1183/13993003.00196-2017

[130] Gibson PG , Yang IA , Upham JW , et al. Effect of azithromycin on asthma exacerbations and quality of life in adults with persistent uncontrolled asthma (AMAZES): a randomised, double‐blind, placebo‐controlled trial. Lancet. 2017;390:659‐668. 28687413 10.1016/S0140-6736(17)31281-3

[131] Douglas LC , Choi J , Esteban‐Cruciani N . Azithromycin treatment in children hospitalized with asthma: a retrospective cohort study. J Asthma. 2020;57:525‐531. 30929521 10.1080/02770903.2019.1590590

[132] Schögler A , Kopf BS , Edwards MR , et al. Novel antiviral properties of azithromycin in cystic fibrosis airway epithelial cells. Eur Respir J. 2015;45:428‐439. 25359346 10.1183/09031936.00102014

[133] Luisi F , Roza CA , Silveira VdA , et al. Azithromycin administered for acute bronchiolitis may have a protective effect on subsequent wheezing. J Bras De Pneumol. 2020;46:e20180376. 10.36416/1806-3756/e20180376 PMC8650811 32130359

[134] Che S‐Y , He H , Deng Y , Liu E‐M . Clinical effect of azithromycin adjuvant therapy in children with bronchiolitis: a systematic review and Meta analysis. Chin J Contemp Pediatrics. 2019;21:812‐819. 10.7499/j.issn.1008-8830.2019.08.014 PMC7389899 31416508

[135] Huckle AW , Fairclough LC , Todd I . Prophylactic antibiotic use in COPD and the potential anti‐inflammatory activities of antibiotics. Respir Care. 2018;63:609‐619. 29463692 10.4187/respcare.05943

[136] Naderi N , Assayag D , Kaddaha Z , et al. Long‐term azithromycin therapy to reduce acute exacerbations in patients with severe chronic obstructive pulmonary disease. Respir Med. 2018;138:129‐136. 29724384 10.1016/j.rmed.2018.03.035

[137] Han MK , Tayob N , Murray S , et al. Predictors of chronic obstructive pulmonary disease exacerbation reduction in response to daily azithromycin therapy. Am J Respir Crit Care Med. 2014;189:1503‐1508. 24779680 10.1164/rccm.201402-0207OC PMC4226018

[138] Uzun S , Djamin RS , Kluytmans JA , et al. Azithromycin maintenance treatment in patients with frequent exacerbations of chronic obstructive pulmonary disease (COLUMBUS): a randomised, double‐blind, placebo‐controlled trial. Lancet Respir Med. 2014;2:361‐368. 24746000 10.1016/S2213-2600(14)70019-0

[139] Clement A , Tamalet A , Leroux E , Ravilly S , Fauroux B , Jais J‐P . Long term effects of azithromycin in patients with cystic fibrosis: a double blind, placebo controlled trial. Thorax. 2006;61:895‐902. 16809416 10.1136/thx.2005.057950 PMC2104771

[140] Aggarwal A , Ghosh A , Gomber S , Mitra M , Parikh A . Efficacy and safety of azithromycin for uncomplicated typhoid fever: an open label non‐comparative study. Indian Pediatr. 2011;48:553‐556. 21555791 10.1007/s13312-011-0093-y

[141] Frenck RW Jr , Mansour A , Nakhla I , et al. Short‐course azithromycin for the treatment of uncomplicated typhoid fever in children and adolescents. Clin Infect Dis. 2004;38:951‐957. 15034826 10.1086/382359

[142] Parry CM , Ho VA , Bay PVB , et al. Randomized controlled comparison of ofloxacin, azithromycin, and an ofloxacin‐azithromycin combination for treatment of multidrug‐resistant and nalidixic acid‐resistant typhoid fever. Antimicrob Agents Chemother. 2007;51:819‐825. 17145784 10.1128/AAC.00447-06 PMC1803150

[143] Vukelic D , Trkulja V , Salkovic‐Petrisic M . Single oral dose of azithromycin versus 5 days of oral erythromycin or no antibiotic in treatment of campylobacter enterocolitis in children: a prospective randomized assessor‐blind study. J Pediatric Gastroenterol Nutr. 2010;50:404‐410. 10.1097/MPG.0b013e3181a87104 19881393

[144] Rustomjee R , Kharsany A , Connolly C , Karim SA . A randomized controlled trial of azithromycin versus doxycycline/ciprofloxacin for the syndromic management of sexually transmitted infections in a resource‐poor setting. J Antimicrob Chemother. 2002;49:875‐878. 12003988 10.1093/jac/dkf034

[145] Macaux L , Zemali N , Jaubert J , et al. Single dose of azithromycin for treatment of patients with asymptomatic rectal chlamydia trachomatis. Acta Derm Venereol. 2020;100:adv00313. 33111150 10.2340/00015555-3675 PMC9309833

[146] Sinha S , Adhikari K , Shankarbabu T , Sinha KT . Azithromycin–the unsung hero in periodontics. IP Int J Periodontol Implantol. 2017;2:1‐4.

[147] Mascarenhas P , Gapski R , Al‐Shammari K , et al. Clinical response of azithromycin as an adjunct to non‐surgical periodontal therapy in smokers. J Periodontol. 2005;76:426‐436. 15857078 10.1902/jop.2005.76.3.426

